# Traceability of On-Machine Tool Measurement: A Review

**DOI:** 10.3390/s17071605

**Published:** 2017-07-11

**Authors:** Unai Mutilba, Eneko Gomez-Acedo, Gorka Kortaberria, Aitor Olarra, Jose A. Yagüe-Fabra

**Affiliations:** 1 Department of Mechanical Engineering, IK4-Tekniker, Eibar 20600, Spain; eneko.gomez-acedo@tekniker.es (E.G.-A.); gorka.kortaberria@tekniker.es (G.K.); aitor.olarra@tekniker.es (A.O.); 2 I3A, Universidad de Zaragoza, Zaragoza 50018, Spain; jyague@unizar.es

**Keywords:** machine tool metrology, temperature, uncertainty, traceability, error sources

## Abstract

Nowadays, errors during the manufacturing process of high value components are not acceptable in driving industries such as energy and transportation. Sectors such as aerospace, automotive, shipbuilding, nuclear power, large science facilities or wind power need complex and accurate components that demand close measurements and fast feedback into their manufacturing processes. New measuring technologies are already available in machine tools, including integrated touch probes and fast interface capabilities. They provide the possibility to measure the workpiece in-machine during or after its manufacture, maintaining the original setup of the workpiece and avoiding the manufacturing process from being interrupted to transport the workpiece to a measuring position. However, the traceability of the measurement process on a machine tool is not ensured yet and measurement data is still not fully reliable enough for process control or product validation. The scientific objective is to determine the uncertainty on a machine tool measurement and, therefore, convert it into a machine integrated traceable measuring process. For that purpose, an error budget should consider error sources such as the machine tools, components under measurement and the interactions between both of them. This paper reviews all those uncertainty sources, being mainly focused on those related to the machine tool, either on the process of geometric error assessment of the machine or on the technology employed to probe the measurand.

## 1. Introduction

“Industry 4.0” represents an initiative for the future development of industrial production [1]. The idea aims to link the manufacturing industry and information technology to make production more flexible, where the flexibility offers the possibility to manufacture customized products through efficient manufacturing processes. As demand fluctuates and batch sizes fall, efficiency in process adjustment and production control operations become crucial. In this context, the importance of measurement technology and its integration in production becomes increasingly significant.

As stated by Imkamp et al. [1], the manufacturing metrology roadmap must address five main challenges: speed, accuracy, reliability, flexibility and holistic measurements. The integration of measurement technology into production processes contributes to cover most of the challenges, where the system works flexibly either for production or measurement purposes. Hence, on-machine tool (MT) measurement shall be applied for machine geometry error monitoring or fast workpiece setup, in-process measurement for flexible manufacturing or post-process measurement for product validation, which leads to a holistic manufacturing system. However, on-MT measurement is influenced by different error sources that are not fully understood yet, which leads to a lack of a metrological traceability chain [2], which in turn means a lack of reliability. In metrology, the accuracy of a measurement is fully understood quantitatively by specifying a measurement uncertainty [1], and this is what is finally aimed at this work, focused on-MT measurement. Thus, a quantitative approach-based error budget is suggested, where MT is the main error source in a MT measurement process. While systematic errors such as geometric errors or touch probe performance are not crucial because they can be compensated, repeatability [2] becomes the major uncertainty contributor. Both temperature variation and MT repeatability itself turn out to be the effects that limit any uncertainty assessment for on-MT measurement. On the other hand, it is also necessary to take into account the contribution coming from the measurand, mainly in large scale metrology applications.

To sum up, this paper contains a review of the existing technologies and methodologies for traceability [2] assessment on a MT measurement. In addition, uncertainty error sources that affect the measurement are analysed in depth and a quantitative approach-based error budget is suggested for determining major error sources.

## 2. Benefits and Limits of on-MT Measurement

**Figure 1:**
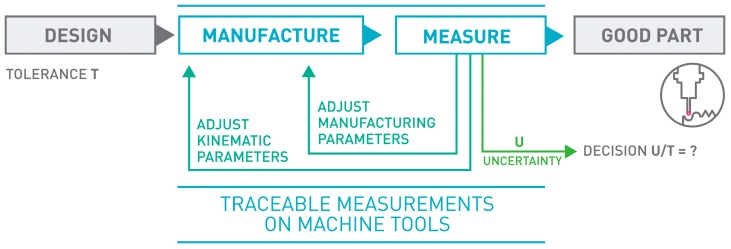
Traceable measurements on machine tools [3].

In order to achieve self-adapting manufacturing processes, dimensional measurements [2] can be employed at different stages of the manufacturing cycle: from the setup and preparation of the MT to be geometrically fitted, to the performance of a final metrology validation of the finished product for final inspection reports and statistical trend analysis. Figure 1 shows the general concept of on-MT measurement. 

**Figure 2:**
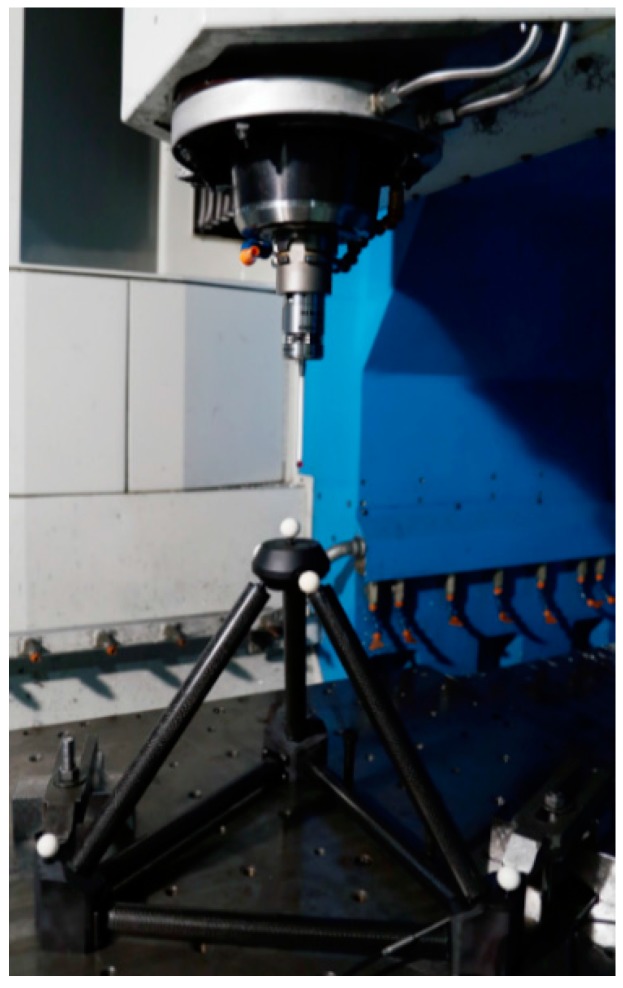
Monitoring machine tool performance.

**Figure 3:**
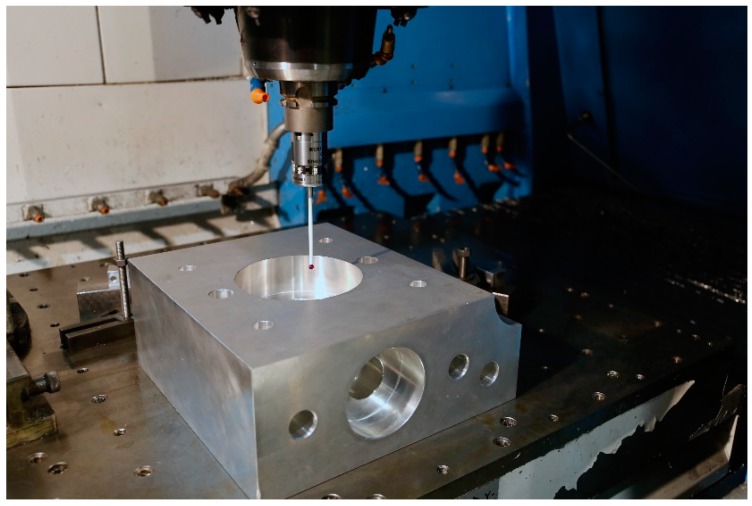
Tactile machine tool probing.

The top four reasons and benefits of on-MT measurement could be listed as follows [4]:

- Monitoring MT Performance: Machine geometry may change during machining operation due to many reasons. By applying an appropriate in-process measurement method with the probe integrated within the MT, geometry changes can be measured. These changes can be monitored to avoid making bad parts and to optimally schedule machine maintenance [4]. Figure 2 shows monitoring of MT performance based on a 3D standard.
- Part Setup: Part cutting programs are created based on an assumed workpiece holding coordinate system. Especially for large parts such as the case for aerospace or large parts manufacturing for automotive applications, this process could take a long time. For small part manufacturing and multi-operation processing, precise part locations could be detected automatically. This would reduce both the setup time and the processing time as parts could be cut from optimally sized blocks [4].
- In-process Measurement: One of the main reasons for performing a metrological measurement [2] of a manufactured part is to provide correction values to manufacturing parameters based on any deviations from the target dimensions found. Having this capability directly on the machine tool allows one to feed back these metrological data to the machine tool controller allowing an automatic flexible manufacturing process. This could be done several times during the manufacturing process, and not just at the end, in order to optimize the part cutting process [4]. Figure 3 depicts a tactile MT probing example.
- Post-process Control: Programming and running a manufacturing machine as if it were a coordinate measuring machine (CMM) for in-process measurement generates complete inspection reports without additional effort. For large part manufacturing, moving the part to an external measuring machine may not even be an option. For mass production, just measuring a few control features would not only generate inspection reports for all the parts but also provide a statistical view of the manufacturing process. In addition, it would help to create historical data monitoring for intelligent process control.

Although on-MT measuring can supply advantages for more flexible and intelligent manufacturing processes, limitations should also be known to make an optimal use of it [5]:

- MT time is more expensive than CMM time: The natural limit of on-MT measurement is given by the time spent on the MT doing measurements. It is known that MT time is more expensive than CMM time, so the measurements done on a MT should clearly add value to the manufacturing process.
- Lack of MT accuracy: MT accuracy is affected by many error sources that change the geometry of the machine´s structural loop. As explained in standard ISO TR 16907 [6], there are different compensation possibilities to enhance the geometric accuracy.
- Lack of MT traceability: Another limitation is given by the lack of traceability of the MT as a CMM. Both machining and measurement operations are performed at the same machine, so if the MT´s geometric error is repeatable, both processes may observe the same geometric error on the measurand.
- Metrology software insufficiencies: Currently software employed in MT is insufficient for metrology purposes. To perform the complex mathematical calculations required for metrology-based real-time decision making, a powerful metrology software needs to be integrated within the manufacturing system.
- Changing environmental conditions: Industrial environments normally suffer from unstable conditions, so it becomes a challenge not just to reduce measurement uncertainties with unfavourable measuring conditions, but to carry out uncertainty assessment for traceable measurement on-MT.

## 3. Converting a MT into a Traceable CMM

In 2010 Schmitt et al. suggested that a large MT should be employed as a comparator to measure the geometry of large scale components during the manufacturing process [7]. Since then, several research works have focused on the idea of converting a MT into a CMM [7,8,9,10,11]. In addition, Schmitt et al. presented a work [12] where the main objective is to define a suitable Maximum Permissible Error (MPE) value for the MT working as a CMM, according to ISO 10360-1 [13]. A tracking interferometer is employed to map the volumetric error of the MT and based on a mathematical model, MPE is determined. Currently, ISO 10360 for MT is a under consensus-based draft development process.

For large scale manufacturing where manufactured parts have to be measured in–situ or in-process, the integration of the measurement process into the MT can improve the process efficiency by preventing the workpiece from being carried to a temperature controlled measuring room. For small and medium size parts, there is a real possibility of achieving finished products on the MT, which offers high product quality, lower manufacturing costs, high productivity and prompt and real-life assessment of product quality [11].

Almost every new machine tool is equipped with a probing system nowadays and offers the possibility to measure product features during or after the manufacturing process. Therefore, machining and measuring processes could take place on the same MT. However, there are some key differences between a CMM and a MT, mainly because a CMM is designed for a measurement purpose and a MT is focused on manufacturing production. The main problem of on-MT measurements is that the machining and measuring operations are performed at the same machine. Therefore, both processes may observe the same geometric error on the measurand, which leads to the point that geometric error of the measurand may not be observed if a geometric error characterization of the MT is not performed before the measurement process. In addition, repeatability can also be a big challenge for a traceable on-MT measurement, where non-controlled shop floor environment becomes a major uncertainty source. Researchers have recognized that environmental temperature has a significant impact on the thermal error of the machine tool and, therefore, on any metrology activity performed on it [14,15,16,17,18,19]. Hence, time- and space-dependent thermal effects become the dominant uncertainty source for the measurement of large scaledevices [8,20].

**Figure 4:**
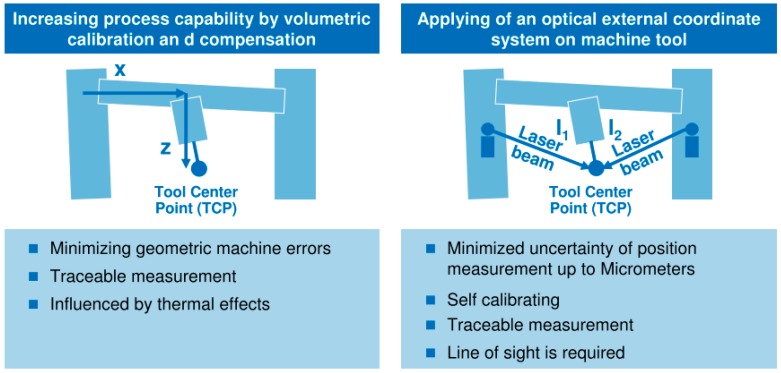
WZL RWTH Aachen approach to convert a MT into a CMM [7].

Schmitt et al. [12] explain two main approaches to convert a MT into a traceable CMM, a scheme of which is shown in Figure 4. The first approach increases the process capability by a volumetric calibration and compensation. It means that a calibration process is done prior to the manufacturing and measuring processes. However, this approach does not ensure that thermal effects do not affect the compensated machine tool. Achievable accuracy can be compared to large CMMs. The second approach applies an external high precision metrological frame to monitor tool centre point (TCP) position in real time. This option requires a line of sight between the measuring tracking interferometers and the TCP, which cannot be ensured when the workpiece is on the MT. Moreover, this option is very sensitive to dirt and dust. The current cost of the solution is very high, since four tracking interferometers are needed at the same time. However, it offers the possibility of being self-calibrating and represents a scalable measuring solution [12].

Currently, the first approach is under research [4], where machine geometric error reduction is of particular importance. Measurement in a shop floor rarely takes place in temperature controlled environment and it means that it is not enough to just measure and compensate geometric errors of the MT, and it must be accompanied by an understanding of how the MT changes. Time- and space- dependent dimensional and gravitational drifts on both MT and the measurand shall be either compensated dynamically or be considered on the uncertainty budget for traceability assessment on-MT measurement.

Although the first approach is being researched in detail, Wendt et al. presented a high accuracy large CMM called M3D3 based on the second approach [21]. In this case, four accurate tracking interferometers are employed for large part calibration directly on-site in production. Schwenke et al. also presented an independent traceable metrology solution for MT measurement based on integrated length monitoring lines on a MT [5].

## 4. Approaches to Determine Measurement Uncertainty on a Machine Tool

Due to the similarity between a CMM and MT, some of the methods for a correct assessment of uncertainty in CMM are adopted for MT. The general guide for a suitable evaluation of measurement data is given in the ISO Guide 98-3: 2008, on the expression of uncertainty in measurement (GUM) [22]. Three different approaches are considered for an uncertainty assessment on a MT dimensional measurement:

### 4.1. Substitution Method Based on ISO 15530-3

The first approach as described in ISO 15530-3 is a method of substitution that simplifies the uncertainty evaluation by means of similarity between the dimension and shape of the workpiece and one calibrated reference part. Moreover, the measurement procedure and environmental conditions shall be similar during evaluation of measurement uncertainty and actual measurement [23]. Due to the similarity requirement between the machined workpiece and the calibrated standard, this approach is very arduous and expensive for large scale metrology. However, it is a suitable approach for small and medium size uncertainty assessment where it is affordable to manufacture and calibrate a reference part for uncertainty assessment purposes.

Across the EURAMET research project Traceable In-process Metrology (TIM), high precision and robust material standards have been developed, not just for mapping the geometric errors of machine tools in the harsh environment of the production floor, but for determining the uncertainties associated with task-specific measurements, such as size, form and position measurements for different geometrical shapes such as sphere, cone, cylinder and plane [24,25,26,27,28,29] by a procedure adopted from ISO 15530-3 [23]. 

### 4.2. Numerical Simulation Based on ISO 15530-4

The second approach is based on ISO 15530-4, a method that is consistent with GUM to determine the task specific uncertainty of coordinate measurements. It is based on a numerical simulation of the measuring process allowed for uncertainty influences, where important influence quantities are taken into account [30]. For that purpose, CMM suppliers, research companies and national metrology institutes (NMI) as PTB and NPL created an uncertainty evaluation software (UES) which is based on Monte-Carlo simulation of the error behaviour of a real CMM [31,32]. In recent years, Virtual gear measuring instrument (VCMM-Gear) and Virtual laser tracker (VLT) have been developed but they have not been integrated into a manufacturer software yet [33]. Nowadays, some research activities are focused on transferring the virtual measuring machines (VMM) concept to virtual measuring processes (VMP) [12,34]. 

### 4.3. Uncertainty Budget Method Based on VDI 2617-11

The third approach is as stated in GUM and VDI 2617-11. In this case, uncertainty evaluation is done based on an uncertainty budget where the budget should comprise the uncertainty sources that affect the measurement process and the correlation between them [35]. Thus, a correct assessment of the measurement uncertainty requires contributions from the measurement system, from the component under measurement and from the interaction between them [8,35]. Currently, this approach is being considered for large scale uncertainty assessment. Schmitt et al. are developing a software-based solution for uncertainty evaluation on large MT measurement [12].

In conclusion, for small batch production, mainly in large scale manufacture, the substitution method is not an affordable solution because a calibrated workpiece similar to the manufactured part is needed. This requirement makes the solution arduous and expensive. Therefore, the uncertainty budget based solution is being adopted for the machine measurement of large workpieces [12]. For serial production, usually for small and medium size components, the substitution method simplifies uncertainty evaluation. Thus, task specific uncertainty can be assessed.

## 5. Uncertainty Error Sources

The uncertainty budget for on machine tool metrology should comprise contributions from the measurement system-i.e., the MT itself (Section 6) with the touching probe (Section 7) and the measuring software (Section 8), from the component under measurement (Section 9) and the interaction between both of them [12,36].

International standard ISO 10360-1 [13] defines a CMM as a measuring system with the means to move a probing system and the capability to determine spatial coordinates on a workpiece surface [37]. Due to the similarity between a CMM and MT, some of the methods for a correct assessment of uncertainty in CMM are adopted for MT. However, there are some key differences between a CMM and a MT, mainly because a CMM is designed for measurement purpose and a MT is focused on manufacturing production. For that reason, here an error budget approach will be suggested for machine tool measurement uncertainty assessment.

**Figure 5:**
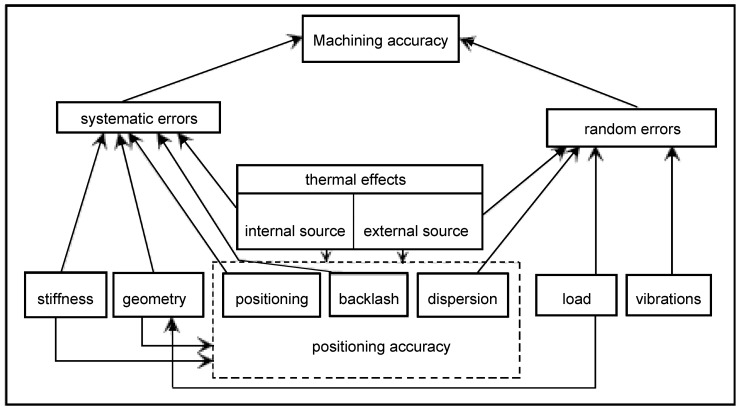
Total error sources of machine tools [40].

As stated by Slocum, MT errors can be divided into systematic errors and random errors [2]. While the former can be measured and compensated, the latter is difficult to predict [38]. Therefore, a machine tool should have three main properties: accuracy, repeatability and resolution [2,39]. Figure 5 represents errors sources of a MT according to the described criteria.

In addition, an error budget is a fast and low cost tool to predict the accuracy and repeatability of a MT [39]. Hence, drawing a comparison between design and measurement purposes, an error budget will be established, where each component will be comprised by:

- Accuracy: Systematic geometric errors of the MT (induced by kinematic errors, static loads and control software), touch probe errors and measuring software errors are considered. The accuracy will mean the systematic error of the MT as a CMM, so it can be characterised and compensated.
- Repeatability: Random error sources that affect the repeatability of the MT. Dynamic loads that affect the MT (such as backlash, forces and thermo-mechanical loads) and environmental influences that affect either the MT or the touch probe are considered. Repeatability will mean the random error of the MT as a CMM, so it is difficult to measure and compensate.
- Resolution: Quality of sensors and quality of control system are considered.

## 6. Error Sources Due to the Machine Tool

### 6.1. Geometric Errors

Either for a CMM or a MT, geometric errors to be considered are relative motion errors between the end effector and the object under measurement. Geometric errors can be measured and compensated when both the MT and the measurement procedure have a high repeatability, so that systematic errors can be reduced and not be considered into the uncertainty budget on a -MT measurement [41].

There are several error sources that affect systematically to the accuracy of the relative end-effector position and orientation [41,42,43,44,45]:

- Kinematic errors: Kinematic errors are errors due to imperfect geometry and dimensions of machine components as well as their configuration in the machine’s structural loop, axis misalignment and errors of the machine’s measuring systems [41,46,47,48,49,50,51,52,53].
- Static loads: In case of static errors, the non-rigid body behaviour has to be considered. Location errors and component errors change due to internal or external forces. The weight of the workpiece and the moving carriages can have a significant influence on the machine’s accuracy due to the finite stiffness of the structural loop [41,54].
- Control software: The effect of the control software on the geometric error of the MT can be considerable. Hence, different speed and accelerations can be applied for a known motion path to make control software errors distinguishable. Anyway, the measurement process is usually executed at small feed speeds, so dynamic forces are usually not considered as an uncertainty contributor on machine tool metrology uncertainty budgets [41].

In practice, the interaction between these effects plays an important role in the overall system behaviour. Here the research is focused on the overall system behaviour, which means the systematic geometric error of the MT [41].

#### 6.1.1. Description of Geometric Errors

Under the assumption of rigid body behaviour, each movement of a machine axis can be described by six components of error, three translations and three rotations. As stated in ISO 230-1, the six component errors of a linear axis are the positioning error, straightness errors, roll error motion and two tilt error motions. For a rotary axis, the six component errors are one axial error motion, two radial error motions, two tilt error motions and the angular positioning error. Moreover, location errors are defined as an error from the nominal position and orientation of an axis in the machine coordinate system. In general, for a linear axis three location errors are considered, while for a rotary axis five location errors are considered [41,55].

#### 6.1.2. Mapping of Geometric Errors

Currently, there are different technologies and measurement methods to characterize all the geometrical errors of a serial kinematic configuration machine. As stated by Schwenke et al. [41] “direct” and “indirect” methods can be distinguished. While direct methods allow the measurement of mechanical errors for a single machine axis without the involvement of other axis, indirect measurements require from the movement of multi-axes of the machine under characterisation.

##### Direct Measurement Methods

As stated by Uriarte et al. [56], direct measurement methods allow to measure component of errors separately regardless of the kinematic model of the machine and the motion of the other axes. Direct measurement can be classified into three different groups according to their measurement principle:

- Standard-based methods, such as straight edges, linear scales, step gauges or orthogonal standards [28,55,57,58,59,60]. Such artefacts contribute also to the uncertainty of the measuring results. This is why their own calibration uncertainty should be as low as possible. However, this is not always reachable, mainly when considering the longest ones and the newest highly accurate machines. Nonetheless, as concluded by Viprey et al., most of the existing material standards are developed for CMM calibration, except ball plates, 1D-ball array and telescopic magnetic ball bar [28], which are suitable for MTs.
- Laser-based methods or multidimensional devices, such as interferometers or telescope bars [61,62,63,64]. They are usually applied in order to measure principally the machine positioning properties, because the suitability of the laser wavelength for long length measurements, due to its long-coherence length. The most used is the laser interferometer which, with different optics configurations, allows detecting position, geometrical and form errors.
- Gravity-based methods that use the direction of the gravity vector as a metrological reference, such as levels [55,65].

**Figure 6:**
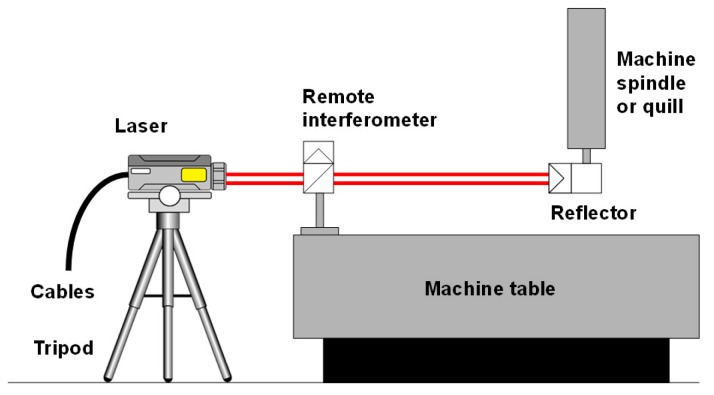
Direct measurement of positioning error through a laser interferometer [67]. (Copyright Renishaw plc. All rights reserved. Image (s) reproduced with the permission of Renishaw.)

While direct measurement methods are frequently employed in small and medium size MT, they are rarely employed for large MT where they are very time-consuming and have strong limitations for a volumetric performance characterization [56]. However, there are some measuring scenarios where direct methods offer advantages compared with indirect methods, such as:

- In small and medium size working volumes direct measurement of an error can approximate the geometric behaviour of a machine tool.
- Specific error motion shall be checked in a very specific line or position. This is depicted in Figure 6.
- Specific verification protocol shall be applied for a machine´s acceptance.
- Iterative “measure and adjust” type of work, which can be needed for component assembly operation.
- Results required in real time.
- High accuracy requirement for a specific application.

For direct measurement of positioning errors, calibrated artifacts (step gauges, gauge blocks, line scales and calibrated encoder system) or laser interferometers are applied as a metrological reference aligned to the axis of interest [41,55,66]. The most accurate/time consuming approach for either short or long machine axis is the use of laser interferometers. Nevertheless, some error sources shall be considered for a correct length measurement [41]:

- Errors in laser wavelength (environmental factors, such as temperature, pressure, humidity and density influence the wavelength compensation).
- Beam deflection shall occur due to temperature changes and gradients.
- Misalignment between interferometer and axis of motion can cause Abbe errors.
- Any movement of the equipment during the measuring process.

Straightness errors of the machine axis can be measured by any of the three measuring principles mentioned before. For large MTs, the most practical way to evaluate straightness is to utilize the direction of the gravity as a reference. Thus, an electronic level is placed on the head of the MT and a reference level is fixed to a non-moving part to distinguish movements of the machine [41]. Measured angle over the stepwise displacement is integrated to get the straightness as a result. However, the linear propagation of a laser interferometer is the industry leading method for large MT straightness measurement. In this case a Wollaston prism acts as a beam splitter and the lateral displacement is calculated from two separate beams that exit the prism at an angle [66,67]. For small and medium size MT, standard based method is commonly applied. Hence, a displacement indicator (capacitance gauges, electronic gauges or material dial gauges) is fixed to the machine head and it detects lateral displacements along the direction of the axis travel [55].

**Figure 7:**
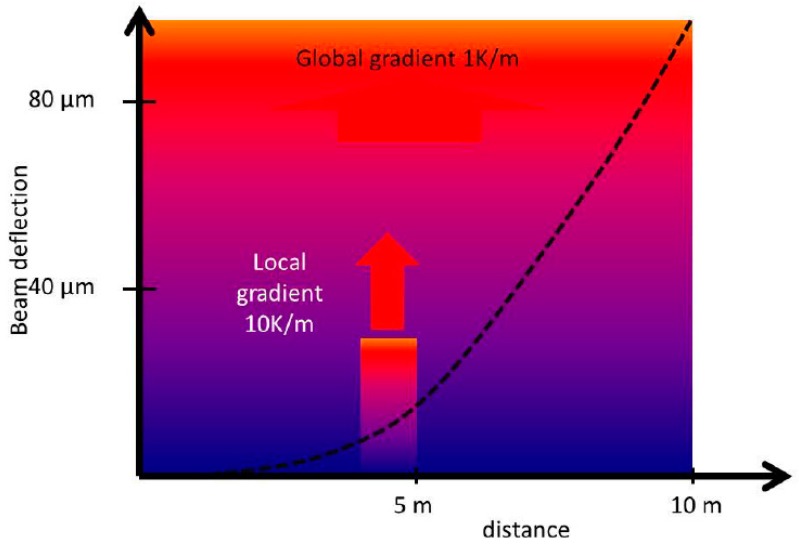
Bending of a straightness reference beam due to local and global gradients [72].

For large MTs and large volume applications, where straightness reference should be long and flat for a long range, a taut wire technique can be used as a straight reference to overcome the limitations of previously mentioned methods [41,68,69]. Even though it has been an extended applied method for very large MTs and applications such as CERN components and assemblies [70], the main reasons why this method is not widely used at present in accurate large MTs are its low accuracy and inefficient data gathering methods [70]. Another approach under investigation for straightness measurements on large volume applications is the use of a laser beam as a straight reference and a position sensitive device (PSD) as a pointing sensor unit. Generally, the use of a laser beam as a straightness reference is highly critical in normal shop floor environment, because local and global temperature gradients as well as air turbulence may have a high influence on the straightness of the beam. Therefore, this method is mostly used for axes length below 1.5 metres, where the influence in most cases is sufficiently small. Also the pointing stability (thermal drift) of optical straightness setups can be a major source of uncertainty [41,71,72]. Figure 7 shows beam deflection according to measuring conditions.

The main approach for squareness measurement in small and medium size MT is to employ granite or ceramic standards with a displacement indicator fixed on the MT head according to the measuring procedure stated in ISO 230-1 [55]. Nevertheless, the main disadvantage of this approach for large MTs is that large and heavy standards are required to verify squareness in large machines. In addition, laser interferometry can also be employed for this purpose but the setup of the laser source and the prism are also very challenging for the squareness error measurement [41].

To measure angular errors in any translation machine axis either the use of electronic levels or laser interferometer based techniques are performed. When applying interferometry, two laser beams are generated with a beam splitter so the angular deviation results in a path length difference of the two beams, but the setup of the measuring system can be very challenging for a correct error assessment in a large axis. In case of electronic levels, they do not depend on an optical path, so they are suitable for the measurement of long strokes in unstable temperature environments. A limitation of electronic levels is that they cannot measure rotations around the gravity vector. For this purpose, in small and medium size axes, an autocollimator is usually employed. A collimated light beam is aligned to the machine axis where a mirror is fixed. The reflected beam travels back to the autocollimator where rotations are measured either visually or through a PSD. However, the unique direct technique to measure the rotation around the axis of motion is based on the use of electronic levels, since an autocollimator or laser interferometer cannot measure this rotation directly [41].

ISO 230-1 [55] describes an affordable method for the calibration of rotary axes. Displacement indicators are fixed to the centre hole of the rotation axis to measure the radial and axial run-out deviations [41]. For the radial and axial error motions three more sensors are needed to be placed on such a way that errors are measured with a linear indicator. If multiple linear indicators are applied, a single measurement combination can be enough for the measurement of the five degrees of freedom [41,73,74,75]. For the positioning error of the rotation axis, the most practical approach is to use laser interferometry combined with a self-centring device and the proper optical optics for angle measurement [76]. This approach is commonly employed in large MTs with a rotary table, due to the measuring range of the solution is around ± 10 and the resolution is better than 0.01 arcseconds [76].

Recently, multidimensional laser interferometers have been introduced to measure more than one degree of freedom (dof) simultaneously. Thus, several error components of a machine axis are determined with a unique measurement system setup through direct measurement methods. This multidimensional measuring solutions offer two main possibilities in the near future. On the one hand, measuring time is reduced to a far extent because different setups and measuring systems are not required anymore. On the other hand, the possibility to be embedded into a MT, where TCP position could be monitored in real time by monitoring six dof of each machine movement at the same time, with several measuring systems performing all at once. 

**Figure 8:**
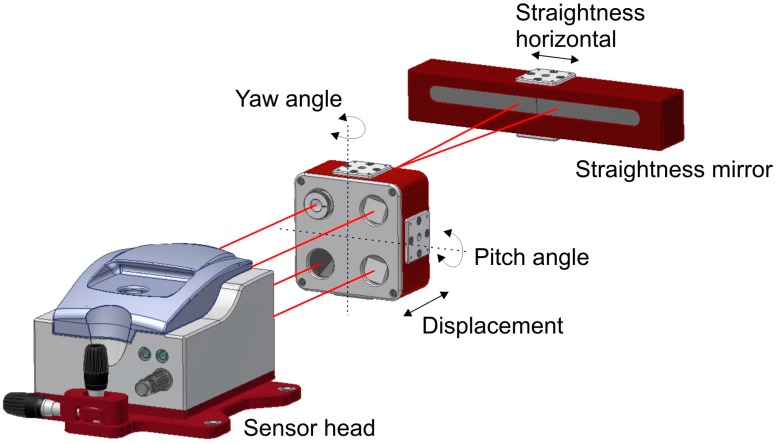
Multidimensional equipment for five dof [78].

In fact, there are two main multidimensional solutions [77,78,79] and the main difference between them is based on the straightness measurement principle. The first solution is a multi-interferometer based solution, where a unique interferometer source is divided into three beams to get a five dof measurement laser interferometer. The second solution employs the laser beam as a straight reference and a PSD as a pointing sensor unit to measure straightness. Therefore, the second option is suitable for small and medium size MT, but not for large MTs [72]. The principle used by the first solution is explained in Figure 8.

As commented before, direct methods are very time-consuming and have strong limitations for large MTs volumetric performance assessment. As explained by Ibaraki et al. [80], for volumetric error compensation, the efficiency of the direct measurement can be a critical issue. In that sense, indirect methods have the advantage of offering fast and reliable volumetric error mapping and compensation possibilities and take less time than direct measurement.

##### Indirect Measurement Methods

Indirect methods produce a global correction of errors and require less time than direct measurement. They are based on the multi-axis movement of the MT under test and can be broken down into two main possibilities [80]:

- Indirect measurement for orthogonal linear axes.
- Indirect measurement for five axis kinematics with rotary axis.

**Figure 9:**
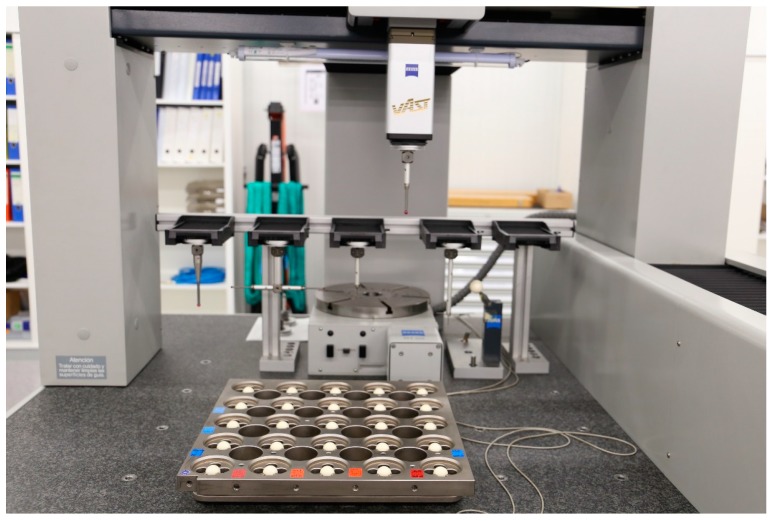
3D artifact for CMM error characterization.

**Figure 10:**
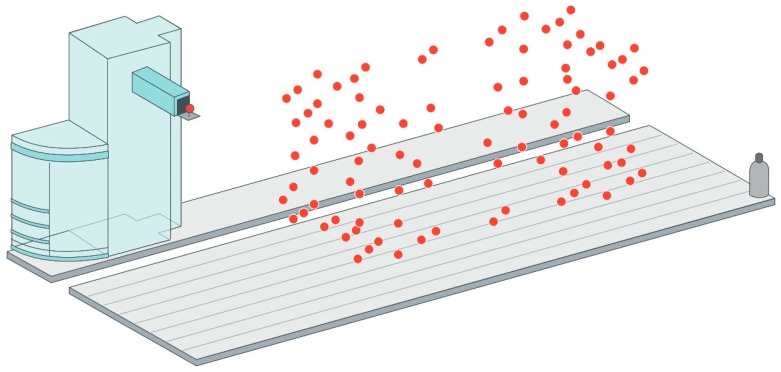
Tracking interferometer and multilateration combination approach.

As stated by Ibaraki et al. [80] there are different procedures and technologies for linear axis indirect characterization:

- Circular tests: The circular test, described in ISO 230-4: 2005 [81] describes a procedure for the characterization of indirect measurement of the geometric accuracy of two orthogonal linear axis. It is usually performed by a ball bar, but it can also be performed by a laser tracer [41] or two dimensional digital scale.
- Diagonal and step-diagonal test: As described in ISO 230-6: 2002 [82], it “allows estimation of the volumetric performance of a machine tool”, but it is not possible to identify 21 geometric error parameter from four body diagonal measurements only. Hence, this test is usually employed for linear scale and squareness error calculation [83]. It is suitable for a fast verification of a MT.
- Measurement of artifacts: The use of calibrated artifacts is widely employed either for MT calibration or CMM calibration. As described by Cauchick-Miguel et al. [60], artifact-based calibration is employed with one dimensional, two dimensional and three dimensional artifacts. The three dimensional artifact is widely employed mainly in CMM calibration for 21 error parameter measurement [84] where pre-calibrated position of spheres are measured by the machine for error characterization. Figure 9 shows a CMM characterization process for virtual coordinate measuring machine error assessment. Since almost every machine tool includes a touch probe nowadays, machine tool builders are looking for fast calibration procedures based on this approach.
- Passive links: Calibrated kinematics of the link mechanism attached to and passively driven by the machine to be measured can be used as a reference [80]. Different link configurations are employed nowadays, either serial links with three orthogonal linear axes or parallel links configurations.
- Tracking interferometer: Tracking interferometers, such as, laser trackers or laser tracers can be employed for indirect error measurement. Laser trackers can directly measure three dimensional position by measuring the distance and direction of a laser beam [80], but angular measurement uncertainty affects the measuring uncertainty of target position and it is rarely employed for MT error measurement. This is the main reason why multilateration based measurement is applied for MT error measurement. In this case, MT position is measured by the distance from at least four tracking interferometers to the target [85,86]. Either laser tracers or laser trackers are usually employed for that purpose. Figure 10 shows a multilateration based scheme, where a tracking interferometer is fixed to the table and the MT or CMM describes a volumetric path through a volumetric point cloud.

For indirect measurement for five axis kinematics with rotary axis, there are also different measuring possibilities:

- Ball bar measurement: As described by Ibaraki et al. [80], there are some standards such as ISO 10791-1:2015 [87] and ISO 10791-6:1998 [88] that define measuring procedures for indirect rotary axis calibration. The calibration of rotary axis location with a ball bar is not solved yet and it remains a challenge.
- R-test: Another approach is to employ R-test to measure relative movements between the machine and the workpiece side. A sphere is fixed to the machine table and a measuring sensor, based on three or more length displacement sensors, is coupled to the machine head [89,90,91]. The measurement consists of a sequence of discrete angles of the rotary table. When moving to the next measurement point the linear axes follow the rotation of the rotary table. At each position the probe head measures the relative displacement of the sphere in X, Y and Z direction simultaneously [91]. Compared to the traditional method that employs “Siemens 996” static cycle to locate a rotary axis in the working volume of a MT, R-test offers the possibility to do static and dynamic measurements [90].
- Measurement of artifacts: As explained for linear axes indirect measurement, any MT has already on machine tool capability. This is why MT probing is being employed for calibration of offset errors of rotary axis [80].
- Machining tests: As explained by Ibaraki et al. [80], MT users are concerned with workpiece´s final accuracy rather than MT accuracy. The National Aerospace Standard (NAS) 979 [92] defines the procedure for a five-axis machining test of a cone frustum, which is widely accepted as a final performance test by machine tool builders.

However, multilateration-based approaches are by far the most used techniques to characterise large machine tools nowadays [7,41,85,86,93,94,95,96,97,98]. The approach relies on interferometric displacement measurements between reference points that are fixed to the machine base and offset points fixed to the machine spindle, near to the TCP [99]. At least four measuring systems are needed for a complete volumetric verification but usually only one measuring device is available, so in practice, multilateration measurements are usually done in a sequential scheme. Thus, machine movements are repeated several times and measurements are taken from different positions. If four measuring devices are available at the same time, simultaneous multilateration avoids some of the limitations of a sequential multilateration, such as total time consumption, MT repeatability requirement and MT drift due to thermal variation during the measuring process.

Several uncertainty sources shall be considered for a complete uncertainty assessment in a sequential multilateration process [95]:

- Volume of the MT.
- Spatial displacement measurement uncertainty of the employed tracking interferometer.
- As stated by Aguado et al. [95], the number of measuring systems to be used and the arrangement of them.
- Repeatability of the measured points does not just depend on the repeatability of the machine itself. As far as the measuring time is extended, environmental influences (e.g., machine shop temperature) generally lead to slow changes of MT temperatures affecting the whole volumetric performance. Therefore, time is a crucial factor.

Currently, three tracking interferometers are being employed on large scale metrology when applying multilateration with different displacement measurement uncertainty:

- Tracking interferometers based on optimized laser trackers. They rely on a high accuracy sphere as optical reference for interferometric measurement. This measurement equipment, called laser tracer [86], was developed by NPL and PTB and commercialised by Etalon AG. It has a spatial displacement measurement uncertainty of U (k = 2) = 0.2 µm + 0.3 µm/m [100]. While laser tracer is a suitable solution for medium and large size MTs, there is a similar solution to the laser tracer, “called laser tracer MT” with a telescopic scheme and employed for maximum measuring volumes of 1 m^3^ [101].
- An Absolute Distance Meter (ADM)-based laser trackers has a spatial displacement measurement uncertainty of U (k = 2) = 10 µm + 0.4 µm/m in its whole working range [102].
- An Absolute Interferometer (AIFM)-based laser tracker has a spatial displacement measurement uncertainty of U (k = 2) = ± 0.4 µm + 0.3 µm/m [102].

The tracking interferometer employed for multilateration shall fit inside the measuring volume in order to execute the measuring procedure. Such a requirement restricts the tracking interferometer to be employed for any size MT. For small size machine tools, the equipment that suitably fits into the measuring volume is the so-called laser tracer MT, it makes use of a metrological beam guiding method of the laser interferometer [101]. For medium and large size MTs, either laser tracers or laser trackers are suitable for the error mapping. However, it should be stated that new laser trackers are portable devices that offer the possibility to be embedded into large manufacturing or measuring systems and they transfer data through an integrated wireless LAN communication [102] which allows to a wireless employment of the acquisition technology.

In this context, different solutions have been developed based on a tracking interferometer and multilateration combination mainly for large MT geometric characterisation, where the volumetric performance of the MT is of special interest: Olarra et al. [97] showed an intermediate approach where linear components of error are measured with a laser tracker based on sequential multilateration. Hence, by combining the data coming from the different measurement systems, multilateration is applied to measure 3D positions with enough accuracy. Once that measured coordinates are calculated, they are compared with nominal positions and geometric errors of the machine tool are deduced from an analytical solution. Additionally, electronic levels are employed for the measurement of the two rotational errors along the two horizontal axes. A self-developed software makes it easier to synchronise data acquisition for both measurement systems and it allows to run the calculation to achieve the aimed volumetric performance of the MT [97]. This approach is similar to the approach described at ISO 10360-2 standard where a calibrated artefact is employed for volumetric error determination [103,104].

Aguado et al. developed an approach where several commercial laser trackers are employed for sequential multilateration measurement [95]. The adopted technical solution is similar to the solution developed by Olarra et al., where laser trackers are applied to acquire information and multilateration is employed to sort out the mathematical issue. The biggest difference is that Aguado et al. do not use electronic levels for the measurement of the rotational errors of the MT.

Schwenke et al. presented a self-developed hardware and software solution for small to large size MT and CMM volumetric characterisation. The commercial laser tracer [100] is employed for point cloud acquisition and from the error of those points and the kinematic model of the machine it is possible to iterate to minimize the global volumetric error of the machine at considered points. For the measurement of angular errors, different orientation offsets on the spindle side are needed, which makes the verification more time-consuming. Nowadays, new configurations and ways of utilisation are appearing for tracking interferometers for MT and CMM geometric error characterization. Etalon AG presented a solution called “Linecal” where several permanently installed measuring lines replace a motorized tracking and device conversion [105].

To sum up, it seems that interferometer-based non-contact measuring technology will guide large scale metrology into traceable machine tool metrology in the near future, mainly because the absolute distance measurements allow an easy handling in industry where purely interferometric length measurements depending on fringe counting are quite demanding due to the need of an unbroken line-of-sight between the measuring instrument and the reflector [8]. However, it shall be remarked that the technology has some key limitations nowadays, such as [8]:

- Thermal and refractive index distortions: The uncertainty of interferometry technique is proportional to the stability of the refractive index of air. Hence, the correct determination of this parameter is of utmost importance for achieving small measurement uncertainties on interferometer based measurements. However, industrial environments normally suffer from unstable conditions, so it becomes a challenge to reduce measurement uncertainties with unfavourable measuring conditions.
- Real time: Real-time coordinate metrology is a requirement for a factory of the future where metrology and manufacture are integrated into a single engineering process that enables 'zero defects'.
- Dimensional traceability to the SI metre: It shall be ensured for any metrology based solution in a factory environment.
- Automation: For a successful integration of the technology into machine and manufacturing processes, wireless and automation capacity shall be improved.

#### 6.1.3. Compensation of Geometric Errors

Traditionally, the majority of MTs have been compensated along lines parallel to the moving axis and centred in the working volume, which is called positioning error mapping and compensation. The ISO230-2 and VDI/DGQ 3441 standards have been widely employed for that purpose and the most common measuring system for error mapping is the laser interferometer [106,107]. However, due to rotational errors of the machine, it is not enough to compensate positioning errors for linear axis if a volumetric accuracy of the MT is aimed. This is the main reason why volumetric compensation was broadly introduced for CMMs fifteen years ago. Volumetric compensation allows not just positioning error compensation, but also compensation of straightness errors, rotational errors and squareness errors. 

Volumetric compensation is now successfully being introduced by main machine tool controller manufacturers on three- or even five-axis machine tools [56]. In general, methodologies based on rigid body kinematics have been proposed [49,108] because the kinematic structure of a MT can be modelled with a kinematic chain and therefore, calculate the position and orientation of the tool in the workpiece coordinate system as the superposition of error motions of each axis [80]. The MT rigid body assumption simplifies the error mapping and compensation because it allows the motion to be implemented by a transformation matrix [109]. Nevertheless, in case of large MTs, due to their size, they suffer from remarkable thermal and mechanical deformations. In order to minimize this effect either on error mapping or compensation, special strategies shall be employed. In compensation, extra compensation factors for the deformation of some parts of the machine, such as column bending and tilt for moving column MT and CMM or table torsion factor for moving table CMMs, are considered [56,72].

Volumetric compensation requires from quantified knowledge of the errors and repeatability from the MT side, as well as time invariant errors. However, changes due to temperature variation play an important role for MT geometry. This is the main reason why limitations must also be known to make a suitable use of compensation. The main limitations are listed next [6,72]:

- Repeatability of the MT: Backlash errors and temperature variation (internal and external) lead to a lack of repeatability. Therefore, long term stability will not be improved.
- Use of long tools: The compensation of orientation requires from three orthogonal rotational axes, which only very few MTs offer. Compensation of angular errors remains a challenge.
- Model conformity: The majority of controllers assume a rigid body model behavior of the machine tool in their compensation models. However, deformations such as column bending and tilt for moving column MTs or table torsion for moving tables CMMs, does not fit to a 21 error model. In these cases, additional parameters shall be included in the compensation model. Therefore, if a model-based compensation is employed, it should be consistent with the machine tool real behavior.

ISO/TR 16907 standard [6] provides information associated with numerical compensation of geometric errors of machine tools. It describes traditional compensation methods such as positioning and straightness compensations and all compensation possibilities within volumetric compensation.

### 6.2. Dynamic Errors

The repeatability of the machine, usually expressed as a standard deviation, is a part of any uncertainty budget and it is mainly affected by dynamic errors. As stated by Slocum, repeatability is difficult to predict and it is often more important to obtain mechanical repeatability, because accuracy can often be obtained by the sensor and control system [39].

There are different error sources that affect the repeatability of the MT working as a CMM: Dynamic loads that affect the MT (such as backlash, dynamic forces and thermo-mechanical loads) and environmental influences that affect either the MT or the touch probe are considered [39,40,41,42,43,44,45].

Between the dynamic loads that affect the MT backlash, dynamic forces and thermo-mechanical loads can be highlighted:

- Backlash: Backlash error is a position dependant error affecting the contouring accuracy. When the axis changes direction from one side to the other, there is a lag before the table starts moving again, that would cause position error- backlash error [110]. Modelling it is challenging, due to multiple sources and complex behaviour. In general, the backlash vector depends on the motion history of all axes. It can result from mechanical play in drives and guideways, cable track forces, and stick/slip effects [72].
- Dynamic forces: Dynamic behaviour of the MT affects the aimed working path. Any varying behaviour, such as, accelerations, varying forces, vibrations or machining forces are hard to measure and compensate. [41,111,112,113].
- Thermo-mechanical errors: Internal and external heat sources combined with different expansion coefficients of machine part materials generate a thermal distortion of the machine’s structural loop which can affect to the accuracy of the measuring process [11,41,114,115,116,117,118]. Expansion coefficient differences may lead to thermal stresses if rules of exact constraint design have not been met carefully.

Apart from dynamic loads affecting the MT, dynamics error sources coming from the touch probe should be also considered. Deviations from the reference temperature of 20 °C lead to thermal expansion or shrinkage of the measuring probe. In addition, temperature variations either inside the workpiece or the stylus, can cause effects like bending. Vibrations may affect the measurement result because it causes a deformation in the metrology loop between probe tip and workpiece. As explained in the previous point, any varying behaviour is hard to measure and compensate, so they contribute directly to the uncertainty of on machine tool metrology [119].

In this scenario, the overall system behaviour is of interest. Some error sources, such as dynamic forces or internal heat sources lead to a fast change of the structural loop that are very hard to measure and compensate [41,111,112,113]. However, there are other error sources such as environmental temperature or simple backlash errors that induce a quasi-static geometric error of the MT that can be monitored and assessed. In fact, quasi-static errors are one of the most important error sources for large scale precision manufacturing [8].

### 6.3. Quasi-Static Error Assessment and Monitoring

The aim of some international research projects, such as “Light controlled factory” or the just finished “Large volume unified metrology for industry novel applications & research (LUMINAR)” and “Traceable in-process dimensional measurements (TIM)”, is to tackle several fundamental issues affecting users of large scale metrology (LSM) equipment and techniques in industrial locations [3,120,121] where non controlled environment affects. In particular, a strong evolution of interferometry-based technology seems to trace the roadmap for the future research of LSM in industrial environment.

Peggs et al. [122] rely on ADM technology as distance measurement principle for future error mapping and monitoring technology. Achievable uncertainty with an ADM (typically 10 mm + 0.4 mm/m) is already being reduced so it is becoming similar to the conventional displacement measuring interferometer (IFM) embedded into laser trackers (typically ± 0.4 µm + 0.3 µm/m) [102]. Consequently, for the built-in displacement device, increasingly absolute distance meters (ADM) are used beside the IFM in commercial laser trackers [8]. While IFM can determine relative distances with accuracies on the nanometer level almost instantaneously, which makes IFM suitable for dynamic measurements, ADM measures absolute distances. However, ADM technology cannot perform dynamic measurements because it must deal with integration times, the time required to perform the operations that determine the target’s position [123].

Schmitt et al. [96] mentioned the extension of the application of interferometry-based technology, which is not only used as a dependent measuring unit but also in multilateration applications, for CMM and MT calibration. An external metrological frame is implemented as a virtual reference based on lengths measured with tracking interferometers. The target positions are calculated using the length measurements with the multilateration principle [21].

**Figure 11:**
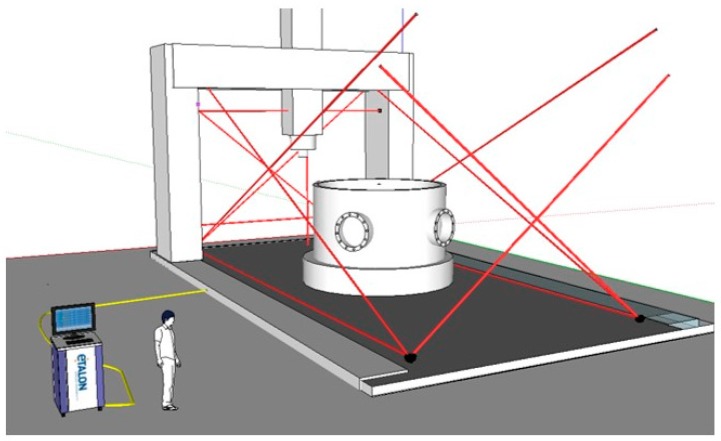
Multiline example on a large MT [125].

Based on ADM technology, multiline technology developed by University of Oxford is a dynamic frequency scanning interferometry (FSI) system scaled up to make many hundreds of measurements for only a small fractional increase in cost compared to laser tracer technology [99], simply by using multiple interferometers whose components are cheap [124]. Despite not having a real time capability (functionality that is under research), this technology allows to monitor large components and structures within an accuracy of 0.5 µm/m. Measurement range is up to 20 metres. It is currently being used in LSM for monitoring of long time stability, deformation by temperature; workpiece weight and foundation drift in many applications [124,125]. An example application can be seen in Figure 11.

As an evolution of the multiline system, a system based on divergent FSI is under development at NPL for real-time coordinate metrology for a factory environment [126]. The measuring system comprises several sensor heads that are placed within the MT volume. Measuring targets, either on the MT or the component under measurement, are defined by spherical retro reflectors. Each sensor head is able to measure absolute distance to multiple targets simultaneously using the mentioned FSI principle. The traceability is ensured through a gas absorption cell embedded into the system and it is used to determine the scale factor for the FSI based distance measurement.

To overcome thermal and refractive index distortions in large volumes, a tracking refractive index compensated interferometer for absolute length measurements, the ‘3D- Lasermeter’, has been developed by PTB and SIOS within LUMINAR European project [121]. The 3D-Lasermeter combines absolute distance measurement by multi-wavelength interferometry, the compensation of the refractive index of air by using the dispersion between two wavelengths, and the tracking capabilities of Laser tracers [8].

**Figure 12:**
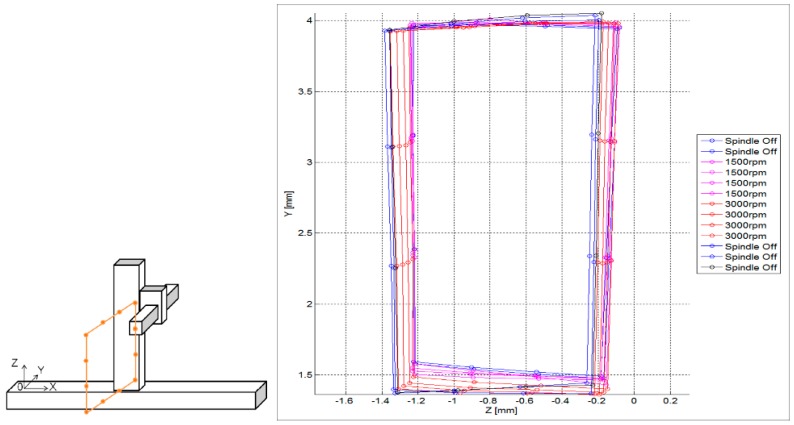
YZ plane measurement and thermal drift assessment on the YZ plane [127].

More practical approaches are presented nowadays. Schwenke et al. present a multilateration-based continuous data acquisition solution (on the fly) where calibration is speeded up significantly by a continuous measurement at constant speed. This option permits to increase the number of sampling points and reduce drastically the measurement time, allowing the measurement of quasi static errors of MTs [100]. However, the measurement process cannot be automated entirely because multilateration is executed in sequence and the device is located by hand. Gomez-Acedo et al. suggest an automatic approach for a fast measurement of thermal distortion on large MTs based on an automatic multilateration measuring procedure [127]. A multilateration scheme is conducted using a single laser tracking device positioned on top of the machine table which moves automatically. As depicted in Figure 12, YZ plane is measured with a sampling period of 20 min during a thermal cycle of 5 h. In addition, Ibaraki et al. [128] present a similar approach where the identification of 2D geometric errors of linear axes by single-setup tests is aimed.

A mobile climate simulation chamber was developed within the mentioned TIM project in order to simulate the variety of influencing factors related to harsh environmental conditions on shop floors [129]. Thus, it is possible to imitate a variety of environments and investigate the behaviour of MT and on MT measurement under these influences.

## 7. Error Sources Due to the Touch Probe 

Probing has become a vital component of automated production processes on machine tools. The probing system should ensure reproducibility during the sensing process even when any adverse influence appears during the process [119,130]. It is necessary to probe the desired point on the real workpiece surface by touching it with a sensing element or by sensing it in a non-contact way [130,131,132]. Often the application will dictate the choice due to limitations in the speed or accuracy of each solution. 

There are two main options when choosing a probing solution: contact or non-contact. There are major differences between both options. The first is that the accuracy of the individual points in contact measurements is higher to that of non-contact measurements. The second is the amount of collected data: non-contact technology can collect millions of sampled points at high speed without touching the workpiece. The third difference is that some surfaces, due to glossiness or transparency, are not suitable for optical measurement and cause special errors [133].

### 7.1. Contact Touch Probe

**Figure 13:**
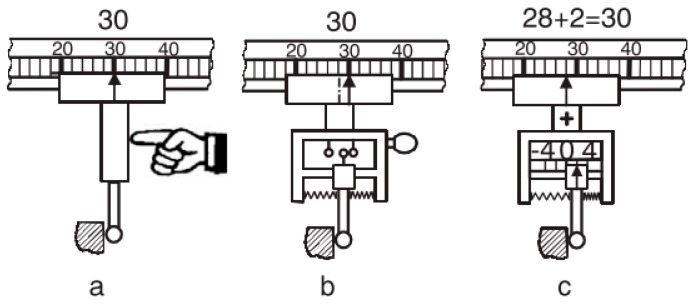
Hard (**a**), touch-trigger (**b**) and measuring (**c**) probe [119].

Contact probes can be divided into two general groups—scanning and discrete—based on the type of data being taken, differences are shown in Figure 13. Discrete probes, or touch trigger probes (TTP), are the most prevalent technology available [134,135]. They have the advantage of being less expensive than some of the other options and are good when fewer data points are needed, such as measurements for position or size [136]. Scanning probes, or analog probes, are continuous contact probes that sense the part as the probe is moved along the expected contour, they are useful in the gathering of high-speed data on a part’s form characteristics [137,138].

#### 7.1.1. Touch Trigger Probe

**Figure 14:**
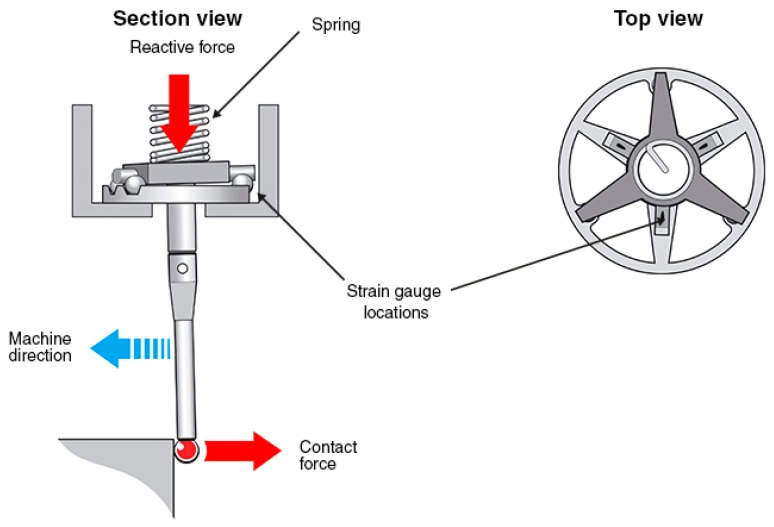
Kinematic resistive probe principle [141]. (Copyright Renishaw plc. All rights reserved. Image (s) reproduced with the permission of Renishaw.)

The two main TTP technologies available for MTs are kinematic resistive probes and strain-gage probes [139,140]. As for kinematic resistive probes, most touch trigger probes utilize a kinematic seating arrangement for the stylus. Three equally spaced rods rest on six tungsten carbide balls providing six points of contact in a kinematic location. An electrical circuit is formed through these contacts. The mechanism is spring loaded which allows deflection when the probe stylus makes contact with the part and also allows the probe to reseat in the same position within 1 μm when in free space (not in contact). Under load of the spring, contact patches are created through which the current can flow. Reactive forces in the probe mechanism cause some contact patches to reduce, which increases resistance of those elements. On making contact with the workpiece (touch), the variable force on the contact patch is measured as a change in electrical resistance. When a defined threshold is reached, a probe output is triggered. The probing sequence is explained in Figure 14.

A number of factors affect the kinematic touch probe measuring performance. From the point at which the stylus ball contacts the workpiece there is bending of the stylus prior to electrical triggering of the probe. This is known as pre-travel. Pre-travel will vary dependent on the length and stiffness of the stylus and the contact force. Pre-travel variation (PTV)-otherwise commonly known as lobing, probe measuring error or roundness measuring error, can affect measurement performance. Lobing occurs because the pivot distance varies depending on the direction in which the contact force acts in relation to the probe mechanism [141]. 

On the other hand, strain gauge probe technology has improved the performance limitations of the kinematic resistive probe technology, mainly because modern compact electronics and solid state sensing have been embedded. Thus, kinematic mechanism retains the stylus and strain gauge technology senses the trigger to acquire the measuring point. As a result, a lower trigger force is needed and uniform pre-travel variation is achieved in all directions [141]. Main differences between both probing technologies are explained in Table 1.

#### 7.1.2. Analog Scanning Probes

Analog scanning probe ensures a permanent and continuous contact between the probe and the component under measurement, so it is particularly suitable for free-form and contoured shaped components as well as for the measurement of large sheet metal assemblies, such as automobile components. Continuous analog scanning (CAS) is a relatively new technology. Its main advantage is the high acquisition speed, which reduces dramatically the measuring time while offers a high density of data acquisition for a full definition of the part’s size, position and shape, enabling completely new opportunities for on-machine tool metrology [119]. Nowadays, there are several CAS systems commercially available for machine tools [143,144].

#### 7.1.3. Factors Affecting Probing Performance

**Figure 15:**
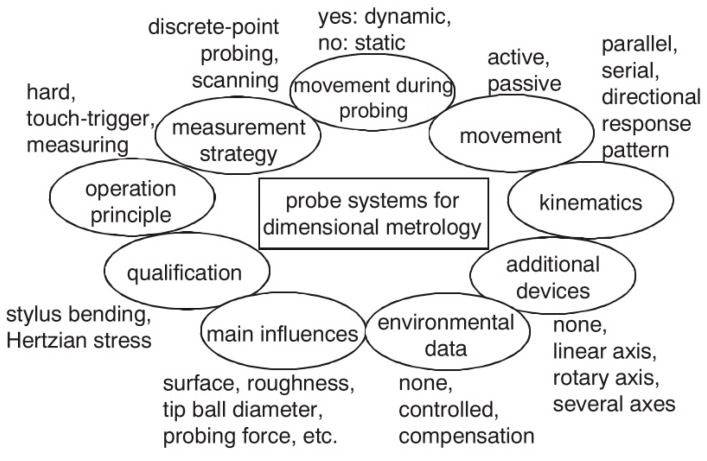
Aspects of probing systems [119].

There are different factors that affect probing performance of touching probe and therefore, their uncertainty must be considered for the MT accuracy assessment when working as a CMM. They are depicted in Figure 15.

**Figure 16:**
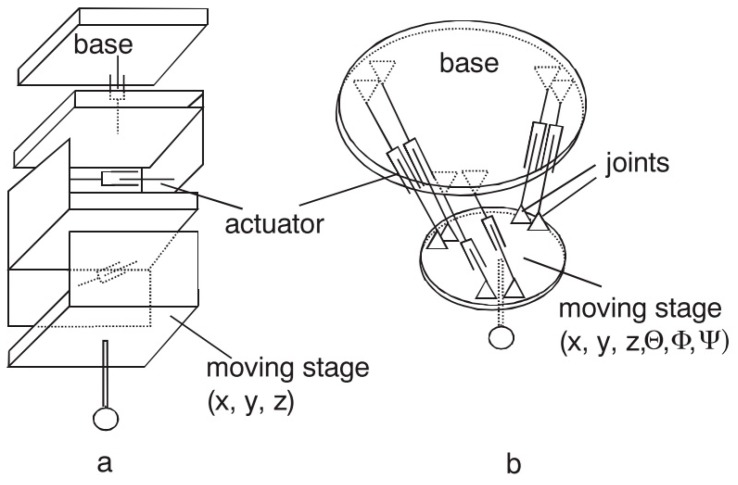
(**a**) serial and (**b**) parallel kinematics probes [119].

- Operation principle: As mentioned in the previous point, contact probes can be broken into two general groups, scanning and discrete, based on the type of data being taken. Based on uncertainty sources, such as pre-travel variation and repeatability, the uncertainty vary according to the contact touch probe selected for the measuring task on the MT.
- Measurement strategy: A disadvantage of discrete-point probing is that it may take a long time to measure a free-form shaped part. If CAS technology is employed a continuous data acquisition is ensured so the acquisition time can be reduced considerably.
- Movement during probing: Static probing is executed while the component under measurement is motionless. However, dynamic measurement involves a component movement during data acquisition. With touch-trigger probes there is no possibility for static measurements as the trigger signal can only be generated during movement [119].
- Movement: The suspension can work either passively, with no actuation, or actively with a spring or electro-mechanical actuator. The active acquisition system offers the possibility to ensure a direction-independent probing force. However, the passive system provides better dynamic properties while probing the component and it is also cheaper [119].
- Kinematics: Probing systems can be mechanically fitted in either a parallel or serial configuration. The configuration influences the static and dynamic behaviour of the probe system, since the size and weight of the probe changes considerably. Serial kinematics comprises several self-independent axes, which are frequently mutually orthogonal. Instead, parallel kinematics configuration involves two axis movement with a coordinate, similar to a hexapod structure [145,146]. Serial and parallel kinematics probes are shown in Figure 16.
- Directional response pattern: A probing system can show varying directional sensitivity response [147,148]; mainly affected by asymmetric arrangement of sensors, asymmetric moment of inertia of stylus, tip ball form error or direction dependent sensitivity of sensors [37]. The effect of direction dependent sensitivity has the result that the same displacement of the tip ball leads to different output signals dependent on the direction of the displacement [149]. However, a correct behaviour characterisation offers the possibility to compensate this anisotropic effect through the control software [150,151,152,153,154].
- Environmental influences: The variation of environmental influences affects every metrology measurement. Consequently, it shall be considered as a part of the repeatability of the MT as a CMM.
- Cleanliness of the Surface: The cleanliness of the surface and the tip ball directly affect the measurement result. Therefore, a clean environment helps to uncertainty reduction on the probing process. In addition, if measurement is executed during the machining process, swarf could seriously influence the probing result. In fact, every effect is related to the probing force. If the probing force is near zero and soft surface contaminations (e.g., oil film) are probed, the signal to noise ratio of the probing system will decrease because of attenuation, which can make a reliable surface detection impossible [119].
- Tip ball: It is the contacting element between the MT and the component under measurement, so it is of utmost importance to characterize its position with the lowest uncertainty. The corrected measured point is achieved by correcting the tip ball centre point by adding a tip correction vector of the length of tip ball radius in the direction from the centre point to the probed point [155]. The radius value of the tip ball is measured during a specific measuring process, called qualification procedure of the probing system [156]. If the probing direction is needed for the coordinate correction process, it can be calculated from the probing system, by interpolation (from at least three probed points in the neighbourhood of the surface point) or by estimation (from e.g., CAD model). Usually real surfaces show, in addition to long-wave form deviations, random short-wave deviations known as roughness [157]. For such a surface the measured geometric properties represent a superposition of measurand and touching element [158] leading to a non- linear mechanical filtering effect. This filtering effect has a characteristic similar to a low pass depending on the tip ball diameter, because a smaller tip ball can penetrate smaller roughness valleys than a bigger ball. Because of this effect one gets for measured features different parameter values (size, position, form deviation) dependent on the diameter of the tip ball. As the measurement result is a superposition of tip ball and surface geometry, also form deviations of the ball directly lead to measurement errors. Thus it is necessary to use a tip ball of negligible form deviation compared to the required measurement uncertainty [119].
- Probing force: The probing force not just causes a bending of the stylus, but also has an effect on the elastic deformation of surface and tip ball due to Hertzian stress. Hertzian stress is the elastic deformation of two bodies touching each other [159]. The extent of deformation is dependent on the materials, micro and macro geometrical forms and the force. The effect of elastic deformations can be compensated to a certain extent by the probing system qualification process.
- Wear of tip ball, plastic deformation and wear of the workpiece surface: Wear and plastic deformation may happen during the probing process. This happens because there are some parameters such as probing force or hardness of contact surfaces that affect to the process. Hence, there are three main effects that cause bad probing results (1) Plastic deformation: Roughness peaks [160] of the workpiece at the probed points may be considered as wear of the workpiece surface [161]. The compressive strength of the workpiece material can be exceeded even by the small probing force because of the very small contact area between tip ball and roughness peak leading to high pressure. It affects the appearance of the probed surface (2) Wear of tip ball can occur during the scanning measuring process on a hard rough surface (3) Materials of tip ball and workpiece interact. It may occur that microscopic small particles break out of the surface due to local welding effects. Under normal circumstances, very little pick-up occurs [119].
- Probing system qualification: The position of the tip ball centre point related to the reference point of the probing system, the radius of the tip ball and the lobing error must be characterised to perform low uncertainty measurements [162,163]. These parameters are determined by a measuring procedure called probing system qualification.

ISO 230-10 [164] specifies test procedures to evaluate the measuring performance of contacting probing systems taking into account many of the factors affecting probing performance here presented. Its scope is limited to probing systems used in a discrete-point probing mode, integrated with a numerically controlled machine tool. It does not include other types of probing systems, such as those used in scanning mode or non-contacting probing systems. As this standard explicitly indicates, it does not address the evaluation of the performance of the machine tool, used as a CMM, since such performance evaluation involves traceability issues and is strongly influenced by machine tool geometric accuracy.

### 7.2. Non-Contact Touch Probe

The availability of non-contact 3D data capture systems capable of acquiring dense geometric data from complex surfaces has increased considerably over the past ten years [165]. Optical non-contact inspection techniques have revolutionized CMM inspection applications in the last decade, due to the cost and coverage of the technology. Nevertheless, a very small percentage of applications with non-contact measurement are already established, especially in robot and machine tool industry [166].

In this scenario, where a few approaches of non-contact technology integration are known, Karadayi presented a blue light laser sensor integration within a five axis machine tool, explaining sensor integration and calibration [167]. The laboratory for machine tools and production engineering of the RWTH Aachen University is also exploring the possibility to integrate non-contact sensors into MTs. Hence, de Moraes et al. integrated a 2D laser into a machine tool for an in-process 3D measurement [168].

**Figure 17:**
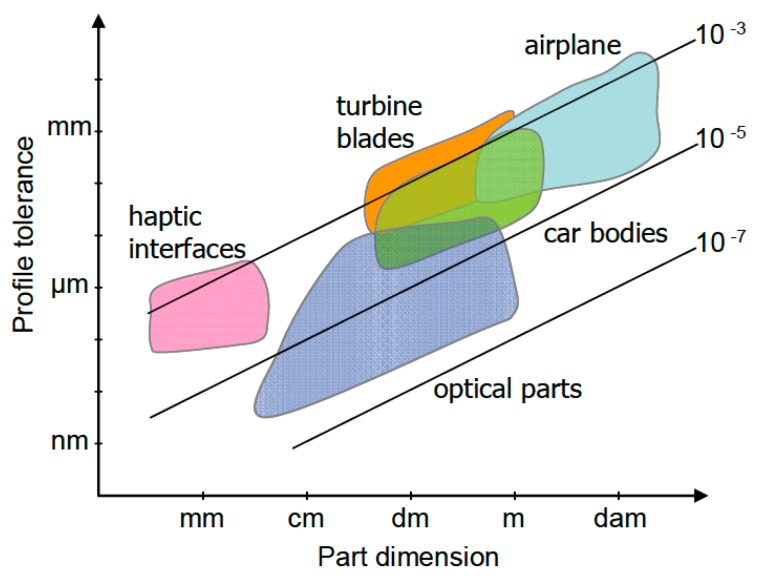
Typical values of tolerances vs. dimensions for most common free form shaped parts [170].

In the manufacturing industry, there is an increasing need to measure accurately 3D shapes. Freeform shaped parts are of great interest in many applications, either for functional or aesthetical reasons. Their relevance for industry is well-known in the design and manufacturing of products having complex functional surfaces [169,170,171,172,173,174,175,176]. These parts are important components in industries such as automotive, aerospace, household appliances and others. Figure 17 shows measuring requirements for most common free form shaped parts.

**Figure 18:**
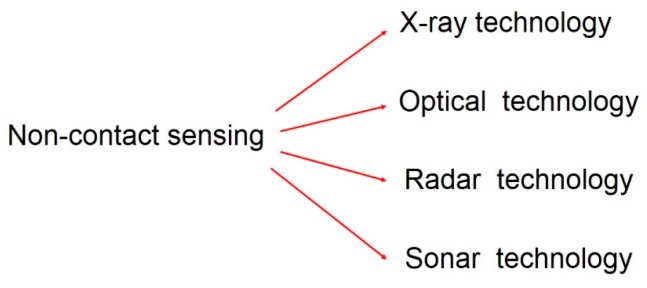
Non-contact sensing technology map.

Currently there is a wide variety of 3D optical sensing techniques that can be potentially integrated into machine tools to verify the geometry of a manufactured part on a machine tool measurement. Figure 18 comprises a non-contact sensing technology map.

**Figure 19:**
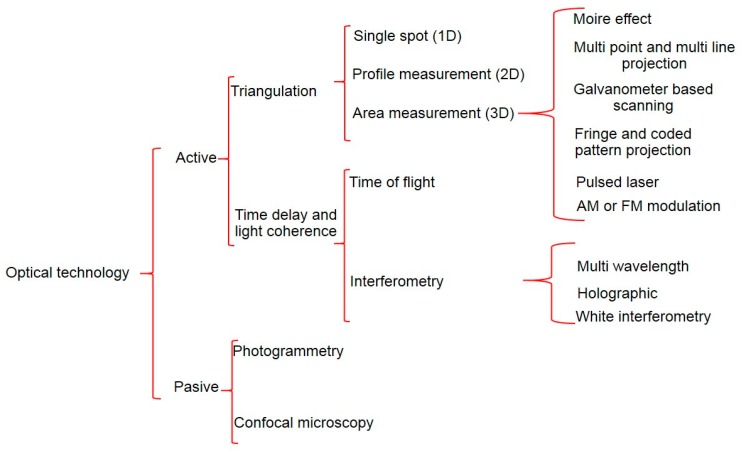
Optical sensing technology map [176].

According to CMM non-contact measurement, optical technology offers the greatest potential for a non-contact measurement on machine tool. Figure 19 shows optical non-contact 3D data capture systems map [176].

Considering the usage of CMM-based inspection by tactile probes and the non-contact optical triangulation systems, it seems that machine tool sensing roadmap will follow the CMM current scenario. Hence, triangulation-based technology is prone to be integrated into MT in the near future complementing the usage of tactile probes in MTs.

### 7.3. Factors Affecting Non-Contact Probing Performance

Additional error sources may appear when using an optical measuring system on a freeform object. The surface characteristic itself dominates the uncertainty of the acquisition process, therefore its variation in terms e.g., of local curvature may add uncertainty. Other common errors are also induced by: the slope of the surface (which may produce direct reflections to the detector), volume scattering (e.g., for plastic material), or an inhomogeneous surface texture. Secondary reflections, specular reflections, volumetric scattering, colour transitions, or ridges left by machining, may lead to gross systematic measuring errors [177,178,179]. 

Post-processing operations of measured data may add further uncertainty. The main difference between discrete and dense point acquisition is the amount and destination of the acquired data. For touch probes the acquired points belong to a single feature, while in scanning acquisition mode the system has no knowledge on which surface or feature the collected points reside. This circumstance is called segmentation and could be the main difference between contact and non-contact technology [180,181]. In fact this is very much like the fundamental problem in computer vision [182,183]. 

## 8. Error Sources Due to the Measuring Software

To perform the complex mathematical calculations required for metrology-based real-time decision making, powerful metrology software needs to be integrated within the manufacturing system. Because the system is expected to function by itself without human interaction, it also needs to work autonomously within the manufacturing process. The following characteristics are required from a software program to truly make a machine tool function similar to a CMM [4]:

- Offline programming: A computer-aided manufacturing (CAM)-style programming environment with good machine tool virtual modelling, simulation capabilities, automatic path generation with collision avoidance, and complete geometrical fitting and tolerancing functionality is required. Programming languages such as DMIS also allow interfacing and collaborating with CMMs for efficient programming.
- Bi-directional interface: A direct and bi-directional interface is a must to analyse data in real time as soon as the measurement of a feature is completed. The calculated metrology characteristics are used as a part of the on-the-fly decision making and written back to the machine tool controller as a part of the adaptive cycle.
- Ability to handle high-density point cloud data: When interfacing with a laser to measure large parts, very large amounts of data will be gathered. The software, in addition to offering a live interface with the machine tool, must also be able to handle the display and interaction with such data.
- Geometric feature extractions: For on-machine geometrical feature measurements and geometric dimensioning and tolerancing (GD&T) applications, an automatic feature extraction is necessary. Most point cloud systems today are offline and need operator interaction to calculate the required features. An on-machine measurement software that will interface with a laser system should also have a robust automatic feature extraction capability.
- Ease of operation: The measurement program must be integrated into the machining centre similar to any other machining program. This allows the measuring to be integrated as a part of manufacturing cycles and can be automatically started by itself. A G-Code NC program is created by post-processing the DMIS measurement routine and resides in the controller.

## 9. Error Sources Due to the Measured Object

Measurement processes are strongly influenced by the measurement systems and especially for large-scale components, by the object itself under measurement. Temperature fluctuations, either in the environment or during the machining process brings to temperature gradients that sensibly influence the geometry of the part, making a significant contribution to the measurement uncertainty. In addition, gravity affects the geometry of the component under measurement. These influences are evident during the manufacturing process of the component, but mainly when doing on-machine tool metrology [8]. 

Component temperature variations comprises a significant uncertainty source for on-MT metrology. The uncertainty increases proportionally with temperature differences and component size. Therefore, for large components measured in a thermally unstable production environment, thermal effects can represent a high percentage of the total measurement uncertainty [8,14,16,114,184,185].

Another heat source is the machining process, which creates a transient and non-homogeneous temperature distribution inside the component. Complex or asymmetric workpieces with different wall thicknesses or materials enhance this thermal inhomogeneity. The heat inside the component affects its characteristics (shape, position and size) when compared to their thermal reference state at 20 °C [8].

Additionally, all the geometric measurements done on earth suffer from gravitational deformations. These elastic deformations depend on the positioning and orientation, the material characteristics and the geometry of the component. Moreover, due to variability on the clamping operation during the machining process, object suffers from varying gravity deformations that affect a potential on-machine measurement during the machining process.

When it comes to the object under measurement, quasi-static errors are not as important as they are for large measuring systems, but it is crucial to determine the behaviour of the component according to a specific temperature and gravitational influences at the moment when measurement is executed [8].

To undertake the necessary modelling to understand and predict how large measurand behave under specific thermal and gravitational conditions, FEM software is widely used. It should be noted that any computational method that can accept temperatures and gravitational forces as a load condition to calculate localized displacements could be applied for such an application [186]. 

The first step is to define with high accuracy the boundary and initial conditions of the simulation. In addition, temperature related information should be characterized, such as, environment temperature information and initial temperature distribution. If the temperature of the part is homogeneous and it is being measured during the manufacturing process, a numerical compensation may be employed for numerical compensation. However, inhomogeneous temperature distributions are difficult to compensate and it should be assigned to the measurement uncertainty [187]. On the other hand, gravity related influences shall be added to the simulation. Information about fixtures that locate and clamp the component on the machine table, clamping orientation related to the gravity vector and detailed information about the component (mass and geometry) are achieved generally from the computer aided design (CAD).

The second step is to run the simulation. Simulation results represent compensation values to be applied as input to the measurement software for compensating thermal geometry and gravitational effects to a certain homogeneous reference temperature and position [8]. 

Finally, post processing is done to achieve results that can be viewed and analysed depending upon the requirements of the on-machine measurement to be done. Commercially available FEM software for the compensation of thermal and gravitational effects are listed next: Abaqus, Ansys, Comsol and Nastran [188,189,190].

## 10. Error Budget Quantitative Approach

The aim at this point is to develop a quantitative approach of a simple error budget [39] on the machine tool side where the weighting factor of each uncertainty source can be distinguished. Hence, main error contributors are detected and future research activities are suggested. 

Small and medium size machine tools, from 0.5 m^3^ to 2 m^3^, typically offer a positioning accuracy better than 5 µm and a repeatability around 2–3 µm [191]. However, as stated by Keller at the TIM final workshop [10], the geometry variation of a 630 mm × 730 mm × 860 mm MT between 15–30 °C could be higher. On this experimental study, the positioning error variation is around 20 µm and the perpendicularity error variation is around 8 µm. While position and squareness errors are dominan°t and strong contributors to the varying total geometric error due to temperature effects, straightness and rotational errors are less prone to temperature effects. Table 2 lists a simple error budget where major error uncertainties are described. Temperature effect is the most important error source, unless it is measured and compensated. As demonstrated by Schmitt et al. the uncertainty of a dimensional measurement done on a MT can be around 20–30 µm for a small MT [12].

The most frequent configurations of large machines are based in serial kinematics and three, four or five motions are located at the machine head. Hence, the part is fixed to the table and a heavy slide to move the part is not required. The dominant serial kinematics configurations for large machines are: movable column, gantry and elevated gantry [56]. The typical positioning accuracy of a high-tech large machine tool is around 10–15 µm and repeatability is better than 10 µm [192]. As stated by Kortaberria at TIM final workshop [193], while the positioning error variation of a large MT (6000 mm × 3000 mm × 1500 mm) is around 80 µm, the squareness and straightness error maintain stable. In addition, as stated by Wennemer [194] a very large MT geometry is extremely sensitive to the temperature influence, a length deviation of 300 µm is shown under temperature variation without any length compensation in beam direction and it is reduced to the half with length deviation. Table 3 depicts a simple error budget for a large MT.

One of the most employed tactile probes nowadays is OMP400 from Renishaw. It offers a repeatability better than 0.5 µm and the 3D lobing error is around ± 2 µm for a 100 mm stylus length [130].

## 11. Outlook and Conclusions

Machine tool measurement has the potential to test product characteristics during or right after the manufacturing process, resulting in improved part quality and reduction of waste material. In addition, the reduction of the production time could also be achieved by preventing the workpiece to be transported to a measuring facility. However, the traceability of the measurement process on a machine tool is not ensured yet and measurement data is still not fully reliable for process control or product validation 

On MT measurement, uncertainty should be assessed according to current CMM standards. For serial production, usually in case of small and medium size components, the substitution method based on ISO 15530-3 simplifies the uncertainty assessment by means of using the similarity between the workpiece and the employed standard. The current scenario shows that traceability for on-machine measurement in small size machine tools based on ISO 15530-3 is starting to be introduced as a realistic option [195]. However, for small batch production, mainly in case of large scale manufacture, the substitution method is not an affordable solution due to the size of the part. Thus, uncertainty should be addressed by uncertainty budget solution according to VDI 2617-11.

There are two main error sources, coming from the MT geometric error variation and the measurand that are not fully understood yet, so traceability cannot be ensured. In addition, position and squareness errors are the dominant contributors to the varying total geometric error of the MT due to temperature effects, but they cannot be permanently monitored at the moment. Moreover, the component under measurement makes a significant contribution to the total measurement uncertainty, mainly because of its thermo-mechanical deformation. Errors resulting from the touch probe should be seriously considered for the error budget of small machine tools, while for large machine tools it represents a minor error source.

The future scenario seems to trust in technology based on interferometry to tackle current limitations. A strong evolution of the technology seems to trace the roadmap for the future research of error mapping and monitoring in industrial environment. ADM technology, which offers absolute measurements, is getting affordable and it is already being used in commercial devices, such as laser trackers, for displacement measurement, besides IFM. As soon as ADM technology achieves IFM uncertainty level and performs real time measurements, a new scenario will arise. It means that MTs and components under measurement could be temperature independent systems where any variation could be recorded by ADM technology in absolute scale and therefore, traceability assessment could be done any time. Following this trend of using technology based on interferometry, some facilities have already gone further by applying technologies like the absolute multiline, in the case of NAMRC [72] or the Productive Process Pyramid^TM^ concept [196] as a full approach to control the process from assessing the machine, through pre-production checks and pre-finishing probing to post-production measurement and Statistical Process Control. These approaches can be applied on some specific cases to have a good understanding of their uncertainty and traceability through interferometry, but they cannot be considered yet as general solutions to ensure the traceability of the measurement process on a machine tool.

In spite of all these advances, many challenges still remain, ranging from the need of technology development to the complete knowledge of the error sources that affect the on-MT measuring process. In addition, a complete system of standards supporting the machine integrated traceable measuring process is needed.

## Figures and Tables

**Table 1:** Comparison table between kinematic and strain gauge probing technology [142].

	Kinematic Resistive Probe	Strain Gauge Probe
**Pros**	Simple mechanism Low mass (so low inertia at the triggering instant) Cost-effective Easy to retrofit to all types of CMM	Improved repeatability Low and almost uniform pre-travel variations in all directions More accurate measurements Low bending deflection (leading low hysteresis) Low trigger force Support much longer styli
**Cons**	Directional dependent pre-travel variation Micro-degradation of contact surfaces Exhibit re-seat failures over time Limiting the length of stylus Resistance through the contact elements as the means to sense trigger	Extra mass (filtering circuitry) Expensive

**Table 2:** Error budget for small and medium size MTs.

Error Source	Significance					
	0–10 µm			10–100 µm		100–1000 µm
**Accuracy**						
Machine tool geometry						
Touch probe						
**Repeatability**						
MT repeatability						
Temperature effect						
Other effects						
**Resolution**						

**Table 3:** Error budget for large size MTs.

Error Source	Significance							
	0–10 µm			10–100 µm			100–1000 µm	
**Accuracy**								
Machine tool geometry								
Touch probe								
**Repeatability**								
MT repeatability								
Temperature effect								
Other effects								
**Resolution**								

## References

[1] Imkamp D., Berthold J., Heizmann M., Kniel K., Peterek M., Schmitt R., Seidler J., Sommer K.-D. (2016). Challenges and trends in manufacturing measurement technology-the “Industrie 4.0” concept. J. Sens. Sens. Syst..

[2] Balazs A. (2008). International vocabulary of metrology-basic and general concepts and associated terms. Chem. Int..

[3] Physikalisch-Technische Bundesanstalt (PTB). https://www.ptb.de/emrp/ind62-home.html.

[4] Karadayi R. In Process Closed Loop Metrology for Adaptive Manufacturing. Proceedings of the Annual Workshop and Conference.

[5] Schwenke H. Large parts with critical tolerances: Concepts and possible solutions for traceable CMM measurements on machine tools. Proceedings of the Traceable in-Process Dimensional Measurement Final Workshop, Physikalisch-Technische-Bundesanstalt (PTB).

[6] (2015). Machine Tools-Numerical Compensation of Geometric Errors.

[7] Nisch S., Schmitt R. Production integrated 3D measurements on large machine tools. Proceedings of the Large Volume Metrology Conference (LVMC).

[8] Schmitt R., Peterek M., Morse E., Knapp W., Galetto M., Härtig F., Goch G., Hughes B., Forbes A., Estler W. (2016). Advances in Large-Scale Metrology–Review and future trends. CIRP Ann. Manuf. Technol..

[9] Werkzeugmaschinenkolloquium A. (2014). Industrie 4.0: Aachener Perspektiven: Aachen Machine Tools Colloquium.

[10] Keller F. Traceability of on-machine measurements under a wide range of working conditions. Proceedings of the Traceable in-Process Dimensional Measurement Final Workshop, Physikalisch-Technische-Bundesanstalt (PTB).

[11] Schmitt R., Jatzkowski P., Peterek M. Traceable measurements using machine tools. Proceedings of the Laser Metrology and Machine Performance X: 10th International Conference and Exhibition on Laser Metrology, Machine Tool, CMM and Robotic Performance, Lamdamap.

[12] Schmitt R., Peterek M. (2015). Traceable measurements on machine tools-thermal influences on machine tool structure and measurement Uncertainty. Procedia CIRP.

[13] (2000). Geometrical Product Specifications (GPS)-Acceptance and Reverification Tests for Coordinate Measuring Machines (CMM)-Part 1: Vocabulary, ISO 10360-1:2000.

[14] Bryan J. (1990). International Status of Thermal Error Research (1990). CIRP Ann. Manuf. Technol..

[15] Bryan J., Pearson J., Brewer W., McClure E. (1965). Thermal effects in dimensional metrology. Mech. Eng..

[16] Ross-Pinnock D., Maropoulos P.G. (2014). Identification of key temperature measurement technologies for the enhancement of product and equipment integrity in the light controlled factory. Procedia CIRP.

[17] Ross-pinnock D., Mullineux G. Compensating for thermal and gravitational effects in structures and assemblies. Proceedings of the Luminar Workshop.

[18] Postlethwaite S.R., Allen J.P., Ford D.G. (1999). Machine tool thermal error reduction-An appraisal. Proc. Inst. Mech. Eng. Part B J. Eng. Manuf..

[19] Mayr J., Jedrzejewski J., Uhlmann E., Alkan Donmez M., Knapp W., Härtig F., Wendt K., Moriwaki T., Shore P., Schmitt R. (2012). Thermal issues in machine tools. CIRP Ann. Manuf. Technol..

[20] Puttock M. (1978). Large Scale metrology. Handbook of Measuring System Design.

[21] Wendt K., Franke M., Härtig F. Mobile Multilateration Measuring System for High Accurate and Traceable 3D Measurements of Large Objects. Proceedings of the 10th International Symposium on Measurement and Quality Control.

[22] (2008). Uncertainty of Measurement-Part 3: Guide to the Expression of Uncertainty in Measurement (GUM:1995).

[23] (2004). Geometrical Product Specifications (GPS)-Coordinate Measuring Machines (CMM): Technique for Determining the Uncertainty of Measurement-Part 3: Use of Calibrated Workpieces or Standards.

[24] Widmaier T., Kuosmanen P., Hemming B., Esala V., Brabandt D. New material standards for traceability of roundness measurements of large-scale rotors. Proceedings of the 58th IWK.

[25] Acko M., Klobucar R., Milfelener M. Measurement Standard for Monitoring Performance of Machine Tools in Harsh Enviromental Conditions. https://www.google.es/url?sa=t&rct=j&q=&esrc=s&source=web&cd=1&ved=0ahUKEwicwMm-x_bUAhUL7RQKHdlaCWYQFggoMAA&url=https%3A%2F%2Fpublic.ptb.de%2Ffiles%2Fdownload%2F56d6a9f2ab9f3f76468b4658&usg=AFQjCNEuMQ3SfbhtfaTwuP6ASTt9CXcXIA.

[26] Zeleny J., Linkeova I. Design and calibration of free-form standard. Proceedings of the Laser Metrology and Machine Performance XI.

[27] Zeleny J., Linkeova I., Skalnik P. (2015). Calibrated CAD model of freeform standard. Proc. XXI IMEKO World Congress 2015.

[28] Viprey F., Nouira H., Lavernhe S., Tournier C. (2016). Novel multi-feature bar design for machine tools geometric errors identification. Precis. Eng..

[29] Woodward S., Brown S., Dury M., McCarthy M. Producing dimensional transfer material standards for the assessment of workshop machine tool performance. Proceedings of the Euspen’s 16th International Conference & Exhibition.

[30] (2008). Geometrical Product Specifications (GPS)-Coordinate Measuring Machines (CMM): Technique for Determining the Uncertainty of Measurement-Part 4: Evaluating Task-Specific Measurement Uncertainty Using Simulation.

[31] Trapet E., Wäldele F. (1991). A reference object based method to determine the parametric error components of coordinate measuring machines and machine tools. Measurement.

[32] Trapet E. (1999). Traceability of Coordinate Measurements According to the Method of the Virtual Measuring Machine.

[33] Härtig F., Kniel K., Schulze K. (2008). Messunsicherheitsermittlung: Ermittlung Einer Aufgabenspezifischen Messunsicherheit von 3D-Verzahnungsmessungen.

[34] Maropoulos P.G., Guo Y., Jamshidi J., Cai B. (2008). Large volume metrology process models: A framework for integrating measurement with assembly planning. CIRP Ann. Manuf. Technol..

[35] (2011). Accuracy of Coordinate Measuring Machines—Characteristics and Their Checking—Determination of the Uncertainty of Measurement for Coordinate Measuring Machines Using Uncertainty Budgets.

[36] García M. Inspection of Large High Value Components on a Machine Tool Platform. Proceedings of the 3D Metrology Conference (3DMC).

[37] Measurement Good Practice Guide No. 42: CMM Verification. http://publications.npl.co.uk/npl_web/pdf/mgpg42.pdf.

[38] Slocum A. Precision Machine-Design-Macromachine Design Philosophy and Its Applicability to the Design of Micromachines. Proceedings of the IEEE Micro Electro Mechanical Systems.

[39] Slocum A. Fundamentals of Design: Error Budgets. http://web.mit.edu/2.75/fundamentals/FUNdaMENTALs%20Book%20pdf/Precision%20Machine%20Design%20Error%20Budget.pdf.

[40] Ispas C., Anania D., Mohora C. Contribution concerning machine tool acuracy using software methods for geometrical errors compensation. Proceedings of the 15th International Conference on Manufacturing Systems-ICMaS.

[41] Schwenke H., Knapp W., Haitjema H., Weckenmann A., Schmitt R., Delbressine F. (2008). Geometric error measurement and compensation of machines-An update. CIRP Ann. Manuf. Technol..

[42] Hocken R.J. (1980). Machine Tool Accuracy. Technology of Machine Tools.

[43] Knapp W., Matthias E. (1983). Test of the Three-Dimensional Uncertainty of Machine Tools and Measuring Machines and its Relation to the Machine Errors. CIRP Ann. Manuf. Technol..

[44] Soons J. Accuracy Analysis of Multi-Axis Machines. https://pure.tue.nl/ws/files/1717013/400139.pdf.

[45] Weekers W.G., Schellekens P.H.J. (1997). Compensation for Dynamic Errrors of Coordinate Measuring Machines. Measurement.

[46] Abbe E. (1890). Meßapparate für Physiker. J. Instrum. Inf..

[47] Ibaraki S., Sawada M., Matsubara A., Matsushita T. (2010). Machining tests to identify kinematic errors on five-axis machine tools. Precis. Eng..

[48] Dassanayake K.M.M., Tsutsumi M., Saito A. (2006). A strategy for identifying static deviations in universal spindle head type multi-axis machining centers. Int. J. Mach. Tools Manuf..

[49] Donmez M.A., Blomquist D.S., Hocken R.J., Liu C.R., Barash M.M. (1986). A general methodology for machine tool accuracy enhancement by error compensation. Precis. Eng..

[50] Huang T., Whitehouse D. (2000). A simple yet effective approach for error compensation of a tripod-based parallel kinematic machine. CIRP Ann. Technol..

[51] Hocken R.J. (1977). Three dimensional Metrology. Proce. CIRP.

[52] Zhang G., Veale R., Charlton T., Borchardt B., Hocken R. (1985). Error Compensation of Coordinate Measuring Machines. CIRP Ann. Manuf. Technol..

[53] Soons J.A., Theuws F.C., Schellekens P.H. (1992). Modeling the errors of multi-axis machines: a general methodology. Precis. Eng..

[54] Schellekens P., Rosielle N., Vermeulen H., Vermeulen M., Wetzels S., Pril W. (1998). Design for Precision: Current Status and Trends. CIRP Ann. Manuf. Technol..

[55] (2012). Test Code for Machine Tools-Part 1: Geometric Accuracy of Machines Operating under no-Load or Quasi-Static Conditions.

[56] Uriarte L., Zatarain M., Axinte D., Yagüe-Fabra J., Ihlenfeldt S., Eguia J., Olarra A. (2013). Machine tools for large parts. CIRP Ann. Manuf. Technol..

[57] Weckenmann A. Comparison of CMM length measurement tests conducted with different 1D, 2D and 3D standards. Proceedings of the 11th National, 2nd International Scientific Conference Metrology in Production Engineering.

[58] Knapp W., Tschudi U., Bucher A. (1990). Vergleich von Prufkorpern zur Abnahme von Koordinatenmessgeräten.

[59] (2015). Test Code for Machine Tools—Part 7: Geometric Accuracy of Axes of Rotation.

[60] Cauchick-Miguel P., King T., Davis J. (1996). CMM verification: A survey. Meas. J. Int. Meas. Confed..

[61] Kunzmann H., Trapet E., Wäldele F. (1990). A Uniform Concept for Calibration, Acceptance Test, and Periodic Inspection of Coordinate Measuring Machines Using Reference Objects. CIRP Ann. Manuf. Technol..

[62] Pahk H.J.A.E., Kimt Y.S.A.M., Moon J.H.E.E. (1997). A New Technique for Volumetric Error Assessment of CNC MTs Incorporating Ball Bar Measurement and 3D Volumetric Error Model. Int. J. Mach. Tools Manuf..

[63] Zargarbashi S.H.H., Mayer J.R.R. (2006). Assessment of machine tool trunnion axis motion error, using magnetic double ball bar. Int. J. Mach. Tools Manuf..

[64] Knapp W. Machine Tool Testing Methods: Overview over ISO Standards and other Special Tests for Machine Tool Performance Evaluation and Interim Checking. Proceedings of the Metroment.

[65] Belforte G., Bona B., Canuto E., Donati F., Ferraris F., Gorini I., Morei S., Peisino M., Sartori S., Levi R. (1987). Coordinate Measuring Machines and Machine Tools Selfcalibration and Error Correction. CIRP Ann. Manuf. Technol..

[66] Knapp W. Comparison of National and International Standards for Evaluation of Positioning Accuracy and Repeatability of NC Axes. http://www.google.es/url?sa=t&rct=j&q=&esrc=s&source=web&cd=1&ved=0ahUKEwiR67_HyfbUAhXFXhQKHaMdCwwQFggjMAA&url=http%3A%2F%2Fwww.amtonline.org%2Farticle_download.cfm%3Farticle_id%3D63300&usg=AFQjCNGIutSKT3NV_EW-A8bbCbTohPDJWQ.

[67] (2015). The Benefits of Remote Interferometry for Linear, Angular and Straightness Measurements.

[68] Hickman P.A. (1968). Optical tilting viewed in a new light. Laser Focus.

[69] Salsbury J.G., Hocken R.J., Inasaki I. (2002). Taut Wire Straightedge Reversal Artifact. Initiatives of Precision Engineering at the Beginning of a Millennium.

[70] Quesnel J., Durand H.M., Touzé T. Stretched Wire Offset Measurements: 40 Years of Practice of This Technique At Cern. Proceedings of the 10th International Workshop on Accelerator Alignment.

[71] Muralikrishnan B., Sawyer D., Blackburn C., Phillips S., Borchardt B., Estler W.T. (2006). ASME B89.4.19 Performance Evaluation Tests and Geometric Misalignments in Laser Trackers. J. Res. Natl. Inst. Stand. Technol..

[72] Schwenke H. The latest trends and future possibilities of volumetric error compensation for machine tools. Proceedings of the 15th International Machine Tool Engineers’ Conference, IMEC.

[73] (1976). CIRP Unification Document Me Axes of Rotation. Ann. CIRP.

[74] Chapman M.A.V., Holloway A., Lee W., May M., McFadden S., Wall D. (2013). Interferometric Calibration of Rotary Axes.

[75] Estler W.T. (1998). Uncertainty Analysis for Angle Calibrations Using Circle Closure. J. Res. Natl. Inst. Stand. Technol..

[76] Chapman M.A.V., Fergusson-Kelly R., Holloway A., Lock D., Lee W. (2013). Interferometric Angle Measurement and the Hardware Options.

[77] XM-60 Multi-Axis Calibrator; Renishaw. http://www.renishaw.com/en/xm-60-multi-axis-calibrator--39258.

[78] Calibration Interferometer: SP 15000 C Series; SIOS. http://www.sios-de.com/products/calibration-interferometer/.

[79] XD Laser Measuring Solution; Automated Precision Inc. (API). https://www.apisensor.com/products/mth/xd-laser/.

[80] Ibaraki S., Knapp W. (2012). Indirect Measurement of Volumetric Accuracy for Three-Axis and Five-Axis Machine Tools: A Review. Int. J. Autom. Technol..

[81] (2005). Test Code for MTs. Part 4. Circular Tests for Numerically Controlled Machine Tools.

[82] (2002). Test Code for Machine Tools. Part 6. Determination of Positioning Accuracy on Body and Face Diagonals (Diagonal Displacement Tests).

[83] Kruth J.P., Zhou L., Van den Bergh C., Vanherck P. (2003). A Method for Squareness Error Verification on a Coordinate Measuring Machine. Int. J. Adv. Manuf. Technol..

[84] Bringmann B., Küng A., Knapp W. (2005). A Measuring Artefact for true 3D Machine Testing and Calibration. CIRP Ann. Manuf. Technol..

[85] Kenta U., Ryosyu F., Sonko O., Toshiyuki T., Tomizo K. (2005). Geometric calibration of a coordinate measuring machine using a laser tracking system. Meas. Sci. Technol..

[86] Schwenke H., Franke M., Hannaford J., Kunzmann H. (2005). Error mapping of CMMs and machine tools by a single tracking interferometer. CIRP Ann. Manuf. Technol..

[87] (2015). Test Conditions for Machining Centres—Part 1: Geometric Tests for Machines with Horizontal Spindle (Horizontal Z-Axis).

[88] (2014). Test Conditions for Machining Centres—Part 6: Accuracy of Speeds and Interpolations.

[89] Florussen G.H.J., Spaan H.A.M. Static R-Test: Allocating the Centreline of Rotary Axes of Machine Tools. Proceedings of the Laser Metrology and Machine Performance VIII: 8th International Conference on Laser Metrology, Machine Tool, CMM & Robotic Performance.

[90] Florussen G.H.J., Spaan H.A.M. Determining the machine tool contouring performance with dynamic R-test measurements 3D probe head Masterball C-axis. Proceedings of the 12th Euspen International Conference.

[91] Florussen G.H.J., Spaan H.A.M. (2012). Dynamic R-test for rotary tables on 5-axes machine tools. Procedia CIRP.

[92] (1969). Uniform Cutting Test—NAS Series. Metal Cutting Equip- Ments.

[93] Hughes E.B., Wilson A., Peggs G.N. (2000). Design of a High-Accuracy CMM Based on Multilateration Techniques. CIRP Ann. Manuf. Technol..

[94] Weckenmann A., Jiang X., Sommer K.D., Neuschaefer-Rube U., Seewig J., Shaw L., Estler T. (2009). Multisensor data fusion in dimensional metrology. CIRP Ann. Manuf. Technol..

[95] Aguado S., Santolaria J., Samper D., Aguilar J.J. (2013). Influence of measurement noise and laser arrangement on measurement uncertainty of laser tracker multilateration in machine tool volumetric verification. Precis. Eng..

[96] Schmitt R., Peterek M., Quinders S. Concept of a Virtual Metrology Frame Based on Absolute Interferometry for Multi Robotic Assembly. https://hal.inria.fr/hal-01260735/document.

[97] Olarra A., Zubeldia M., Gomez-acedo E., Kortaberria G. Measuring positioning accuracy of large machine tool. Proceedings of the 19th Congress of Machine Tools and Manufacturing Technology.

[98] Brecher C., Flore J., Haag S., Wenzel C. High precision, fast and flexible calibration of robots and larde multi-axis machine tools. Proceedings of the 9th International Conference on Machine Tools, Automation, Robotics and Technology.

[99] Schwenke H. (2015). Accuracy Improvement of Machine Tool via New Laser Measurement Methods.

[100] Schwenke H., Schmitt R., Jatzkowski P., Warmann C. (2009). On-the-fly calibration of linear and rotary axes of machine tools and CMMs using a tracking interferometer. CIRP Ann. Manuf. Technol..

[101] LaserTracer-MT: Perfect Geometries through Volumetric Compensation. http://www.etalon-ag.com/en/products/lasertracer-mt/.

[102] Hexagon Manufacturing Intelligence. http://www.hexagonmi.com/products/laser-tracker-systems/leica-absolute-tracker-at930.

[103] (2009). Geometrical Product Specifications (GPS)—Acceptance and Reverification Tests for Coordinate Measuring Machines (CMM)—Part 2: CMMs Used for Measuring Linear Dimensions.

[104] Trapet E., Aguilar Martín J.J., Yagüe J.A., Spaan H., Zelený V. (2006). Self-centering probes with parallel kinematics to verify machine-tools. Precis. Eng..

[105] Etalon AG Linecal-Automatic Volumetric Calibration. http://www.etalon-ag.com/en/products/linecal/.

[106] (2014). Test Code for Machine Tools-Part 2: DETERMINATION of Accuracy and Repeatability of Positioning of Numerically Controlled Axes.

[107] (1982). Statistical Testing of the Operational and Positional Accuracy of Machine Tools.

[108] Longstaff A.P., Fletcher S., Myers A. Volumetric error compensation through a Siemens controller. Proceedings of the 7th International Conference and Exhibition on Laser Metrology, Machine Tool, CMM & Robotic Performance, Lamdamap 2005.

[109] Duffie N.A., Yang S.M., Bollinger J.G. (1985). Generation of Parametric Kinematic Error-Correction Functions from Volumetric Error Measurements. CIRP Ann. Manuf. Technol..

[110] Liu H., Xue X., Tan G. (2010). Backlash Error Measurement and Compensation on the Vertical Machining Center. Engineering.

[111] Rehsteiner F., Weikert S., Rak Z. Accuracy Optimization of Machine Tools under Acceleration Loads for The Demands of High-Speed-Machining. Proceedings of the 13th Annual Meeting of American Society for Precision Engineering.

[112] Weikert S. When five axes have to be synchronized. Proceedings of the 7th International Conference and Exhibition on Laser Metrology, CMM and Machine Tool Performance.

[113] Weekers W.G., Schellekens P.H.J. (1997). Compensation for dynamic errors of coordinate measuring machines. Precis. Eng..

[114] Bryan J. International Status of Thermal Error Research. http://emtoolbox.nist.gov/Publications/UCRL-1967-50285-InternationalStatusofThermalErrorResearch.pdf.

[115] Van Den Bergh C. (2001). Reducing thermal errors of CMM located on the shop—Floor. Ph.D. Thesis.

[116] Delbressine F.L.M., Florussen G.H.J., Schijvenaars L.A., Schellekens P.H.J. (2006). Modelling thermomechanical behaviour of multi-axis machine tools. Precis. Eng..

[117] Florussen G.H.J. (2002). Accuracy Analysis of Multi-axis Machines by 3D Length Measurements. Ph.D. Thesis.

[118] Gruber R., Knapp W. (1998). Temperatureinflüsse auf die Werkzeugmaschinen-Genauigkeit. Werkstatt und Betried.

[119] Weckenmann A., Estler T., Peggs G., McMurtry D. (2004). Probing systems in dimensional metrology. CIRP Ann. Manuf. Technol..

[120] Muelaner J.E., Maropoulos P.G. (2014). Large volume metrology technologies for the light controlled factory. Procedia CIRP.

[121] National Physical Laboratory (NPL) Large Volume Unified Metrology for Industry, Novel Applications & Research (LUMINAR) EMRP Project. http://projects.npl.co.uk/luminar/.

[122] Peggs G.N., Maropoulos P.G., Hughes E.B., Forbes A.B., Robson S., Ziebart M., Muralikrishnan B. (2009). Recent developments in large-scale dimensional metrology. Proc. Inst. Mech. Eng. Part B J. Eng. Manuf..

[123] Leica Absolute Interferometer, a New Approach to Laser Tracker Absolute Distance Meteres. Leica. http://www.google.es/url?sa=t&rct=j&q=&esrc=s&source=web&cd=1&ved=0ahUKEwihsanj-vPUAhWDXhQKHdUcC8AQFggrMAA&url=http%3A%2F%2Fwww.hexagonmi.com%2F~%2Fmedia%2FHexagon%2520MI%2520Legacy%2Fm1%2Fmetrology%2Fgeneral%2Fwhite-tech-paper%2FLeica%2520Absolute%2520Interferometer_white%2520paper_en.ashx&usg=AFQjCNE1v4rh9xtnLvH9BePSkNh3PgrpNA.

[124] Cabral A., Abreu M. Absolute distance metrology for long distances with dual frequency sweeping interferometry. Proccedings of the XIX Imeko World Congress, Fundamental and Applied Metrology.

[125] Absolute Multiline Technology. Etalon AG. http://www.etalon-ag.com/produkte/absolute-multiline-technologie/.

[126] Hughes B., Warden M.S. (2013). Novel Coordinate Measurement System Based on Frequency Scanning Interferometry. J. Coord. Metrol. Syst. Conf..

[127] Gomez-Acedo E., Olarra A., Zubieta M., Kortaberria G., Ariznabarreta E., de Lacalle L.N. (2015). Method for measuring thermal distortion in large machine tools by means of laser multilateration. Int. J. Adv. Manuf. Technol..

[128] Ibaraki S., Blaser P., Shimoike M., Takayama N., Nakaminami M., Ido Y. (2016). Measurement of thermal influence on a two-dimensional motion trajectory using a tracking interferometer. CIRP Ann. Manuf. Technol..

[129] Berger D., Brabandt D., Lanza G. (2015). Conception of a mobile climate simulation chamber for the investigation of the influences of harsh shop floor conditions on in-process measurement systems in machine tools. Meas. J. Int. Meas. Confed..

[130] OMP400 Optical Machine Probe systems. Renishaw. http://www.google.es/url?sa=t&rct=j&q=&esrc=s&source=web&cd=2&ved=0ahUKEwjQzofc_vPUAhUFRhQKHec8AsoQFgg1MAE&url=http%3A%2F%2Fresources.renishaw.com%2Fen%2Fdownload%2Finstallation-guide-omp400--47616&usg=AFQjCNH8VtRcINKwlpWnhGQu8NZNWn0HRA.

[131] Weckenmann A. Comparability of tactile and optical form measuring techniques with resolution in the nanometre range. Proceedings of the X. International Colloquium on Surfaces.

[132] Yang Q., Butler C. (1996). A 3-D noncontact trigger probe for coordinate measuring machines. Meas. J. Int. Meas. Confed..

[133] Lukacs G., Lockhart J., Facello M. Non-contact Whole-Part Inspection. https://www.cmsc.org/stuff/contentmgr/files/0/2bdcf766d9d5daf6e892c46153c591d3/misc/cmsc2011_thur_gh_0830_lockhart_paper.pdf.

[134] Marsh B. (1996). An Investigation of Diameter Measurement Repeatability Using A Coordinate Measuring Machine And A Multi-Baseline Repeatability Assessment Methodology. Ph.D. Thesis.

[135] Dove J. (2000). Probe qualification and precision coordinate metrology. Manuf. Eng..

[136] (2005). Measurement on CMMs.

[137] Imkamp D., Schepperle K. (2006). The Application Determines the Sensor: VAST Scanning Probe Systems. Innov. Spec. Metrol..

[138] Gage R&R Studies on CMM Accuracy. Hexagon Metrology. http://pcdmiswiki.org/images/0/00/GRR_Studies_on_CMM_Accuracy_Hex.pdf.

[139] Ali S.H.R. (2010). Probing System Characteristics in Coordinate Metrology. Meas. Sci. Rev..

[140] Berisso K., Ollison T. (2010). Coordinate Measuring Machine Variations for Selected Probe Head Configurations. J. Ind. Technol..

[141] (2005). Probing Systems for CNC Machine Tools.

[142] Karuc E. Design of a Touch Triger Probe for a Coordinate Measuring Machine. https://etd.lib.metu.edu.tr/upload/12609112/index.pdf.

[143] (2014). SPRINT High-Speed Scanning System.

[144] Digilog Touch Probes. Novotest. http://www.blum-novotest.com/en/products/measuring-components/digilog-touch-probes/tc63-tc64-digilog.html.

[145] Weckenmann A., Kampa H. Koordinaten messgeraete, VDI-Z. https://de.wikipedia.org/wiki/Koordinatenmesstechnik.

[146] Weckenmann A., Krämer P. Trends in der Koordinatenmesstechnik Multisensorik. http://www.saq.ch/fileadmin/user_upload/mq/downloads/mq_2012_01_weckenmann.pdf.

[147] Aston R., Davis J., Stout K. (1998). A probing question: A customer´s investigation into the directional variability of a coordinate measuring machine touch trigger probe. Int. J. Mach. Tools Manuf..

[148] Chan F.M.M., Davis E.J., King T.G., Stout K.J. (1997). Some performance characteristics of a multi-axis touch trigger probe. Meas. Sci. Technol..

[149] Dobosz M., Woźniak A. (2003). Metrological feasibilities of CMM touch trigger probes. Measurement.

[150] Shen Y.-L., Zhang X. (1997). Pretravel compensation for vertically oriented touch-trigger probes with straight styli. Int. J. Mach. Tools Manuf..

[151] Kishinami T., Nakamura H., Saito K. Three Dimensional Curved Surfaces measurement using Newly Developed Three Dimensional Tactile Sensing Probe. Proceedings of the International Symposium on Metrology for Quality Control in Production (ISMQC).

[152] Moons S., Shen Y.-L. (1998). Errors in probe offset Vectors of multiple orientations in CMM measurements. ASPE Proc..

[153] Ogura I., Okazaki Y. (2003). A Study for Development of Small-CMM Probe detecting Contact Angle. Proc. ASPE Summer Top. Meet..

[154] Yang Q., Butler C., Baird P. (1996). Error compensation of touch trigger probes. Meas. J. Int. Meas. Confed..

[155] Li Y.F., Liu Z.G. (2003). Method for determining the probing points for efficient measurement and reconstruction of freeform surfaces. Meas. Sci. Technol..

[156] Koordinatenmesstechnik. http://www.google.es/url?sa=t&rct=j&q=&esrc=s&source=web&cd=39&ved=0ahUKEwjzxZzJzPbUAhVMPBQKHRZ-A844HhAWCEYwCA&url=http%3A%2F%2Fapi.vlb.de%2Fapi%2Fv1%2Fasset%2Fmmo%2Ffile%2F7491cffd-5777-4e4c-9c0e-9d269cbc33c1%3Faccess_token%3De047cd7a-ea60-44d5-adc2-59fdaa680c67&usg=AFQjCNHkVQl3FKMBBb5LIx2lwqYBr7wCfA.

[157] Uhlmann E., Förster R., Laufer J., Sroka F. (2000). Three-dimensional roughness values for the functional characterization of ceramic surfaces. X Int. Colloq. Surf..

[158] Lonardo P.M., Lucca D.A., De Chiffre L. (2002). Emerging Trends in Surface Metrology. CIRP Ann. Manuf. Technol..

[159] Puttock M.J., Thwaite E.G. (1969). Elastic Compression of Spheres and Cylinders at Point and Line Contact. Natl. Stand. Lab. Tech. Pap..

[160] Uhlmann E., Oberschmidt D., Kunath-fandrei G. 3D-Analysis of Microstructures with Confocal Laser Scanning Microscopy. http://aspe.pointinspace.com/publications/Winter_2003/03W%20Extended%20Abstracts/uhlmann.PDF.

[161] Zahwi S., Mekawi A.M. (2001). Some effects of stylus force on scratching surfaces. Int. J. Mach. Tools Manuf..

[162] Edgeworth R., Wilhelm R.G. (1996). Uncertainty Management for CMM Probe Sampling of Complex Surfaces. ASME Manuf. Sci. Eng..

[163] Aoyama H., Kawai M., Kishinami T. (1989). A New Method for Detecting the Contact Point between a Touch Probe and a Surface. CIRP Ann. Manuf. Technol..

[164] (2016). Test Code for Machine Tools—Part 10: Determination of the Measuring Performance of Probing Systems of Numerically Controlled Machine Tools.

[165] Marshall S., Gilby J. (2001). New Opportunities in Non-Contact 3D Measurement. Natl. Meas. Conf..

[166] Keferstein C., Züst R. (2004). Minimizing technical and financial risk when integrating and applying optical sensors for in- process measurement. Int. Intell. Manuf. Syst. Forum.

[167] Karadayi R. Blue Light Laser Sensor Integration and Point Cloud Metrology. https://www.qualitydigest.com/inside/cmsc-article/081816-blue-light-laser-sensor-integration-and-point-cloud-metrology.html#.

[168] Paulo de Moraes J., Peterek M. (2012). Integration of a Laser Scanner into a Machine Tool for an in-Process 3D Measurement. Master’s Thesis.

[169] Streppel A.H., Lutters D., ten Brinke E., Pijlman H., Kals H.J. (2001). Selective measuring of freeform surfaces for quality control and selective maintenance of bending tools. J. Mater. Process. Technol..

[170] Savio E., Chiffre L. (2007). De; Schmitt, R. Metrology of freeform shaped parts. CIRP Ann. Manuf. Technol..

[171] Brinksmeier E., Preuss W. Diamond Machining of the 3 m Reflector of the KOSMA Submillimeter Telescope by a Single-Point Fly-Cutting Process. https://www.tib.eu/en/search/id/BLCP%3ACN019396568/Diamond-Machining-of-the-3m-Reflector-of-the-KOSMA/.

[172] Brinksmeier E., Riemer E., Glabe O. Fabrication of Complex Optical Elements. https://mlecture.uni-bremen.de/ml/index.php?option=com_content&view=article&id=172&template=ml2.

[173] Katahira K., Ohmori H., Yoshida K., Kasuga H., Hirai S. Elid Grinding Effects on Fabrication of Advanced Ceramics Components Riken. http://aspe.net/publications/Annual_2009/POSTERII/01GRIND/2941.PDF.

[174] Klocke F., Dambon O. Precision Machining of Glass for Optical Applications. Proceedings of the International Workshop on Extreme Optics and Sensors.

[175] Savio E., De Chiffre L. Inspection of free form functional surfaces on fan blades. Proceedings of the PRIME 2001 International Conference.

[176] Beraldin J.-A., Gaiani M. (2005). Evaluating the Performance of Close-Range 3D Active Vision Systems for Industrial Design Applications. Videometrics VIII Int. SPIE Electron. Imaging.

[177] Carmignato S. (2005). Traceability of Coordinate Measurements on Complex Surfaces. Ph.D. Thesis.

[178] Carmignato S., Neuschaefer-Rube U., Schwenke H., Wendt K. Tests and artefacts for determining the structural resolution of optical distance sensors for coordinate measurement. Proceedings of the 6th International Conference European Society for Precision Engineering and Nanotechnology.

[179] Sansoni G., Carmignato S., Savio E. (2004). Validation of the measurement performance of a three-dimensional vision sensor by means of a coordinate measuring machine. Conf. Rec. IEEE Instrum. Meas. Technol. Conf..

[180] Bartscher M., Hilpert U., Neuschaefer-Rube U. (2007). Industrielle Computertomographie auf dem Weg zur Koordinatenmesstechnik. PTB Meet..

[181] Bartscher M., Neukamm M., Hilpert U., Neuschaefer-Rube U., Härtig F., Kniel K., Ehrig K., Staude A., Goebbels J. (2010). Achieving Traceability of Industrial Computed Tomography. Key Eng. Mater..

[182] Sonka M., Hlavac V., Boyle R. (1999). Image Processing, Analysis, and Machine Vision.

[183] Chen X., Golovinskiy A., Funkhouser T. Benchmark for 3D Mesh Segmentation. Proceedings of the Siggraph.

[184] Mian N.S., Fletcher S., Longstaff A.P., Myers A. (2013). Efficient estimation by FEA of machine tool distortion due to environmental temperature perturbations. Precis. Eng..

[185] Maier T., Zaeh M.F. (2012). Modeling of the thermomechanical process effects on machine tool structures. Procedia CIRP.

[186] Klocke F., Lung D., Puls H. (2013). FEM-modelling of the thermal workpiece deformation in dry turning. Procedia CIRP.

[187] Muelaner J.E. Coping with Thermal Expansion in Large Volume. http://projects.npl.co.uk/luminar/publications/luminar-gpg-thermal-expansion-lvm.pdf.

[188] Adams V., Askenazi A. (1999). Building Better Products with Finite Element Analysis.

[189] Finite Element Analysis: Theory and Application with ANSYS. Moavani. http://ftp.demec.ufpr.br/disciplinas/TM310/livro/Finite%20Element%20Analysis,%20Theory%20and%20application%20with%20ANSYS,%20.pdf.

[190] Nakasone Y. (2006). Engineering Analysis with ANSYS Software.

[191] Makino Vertical Machining Center-ps-Series-vmc. https://www.makino.com/vertical-machining-centers/ps-series-vmc/ps-brochure-(electronic).pdf.

[192] KAIROS Machine Tool. ZAYER. http://www.zayer.com/en/product/movingcolumn/kairos/25.

[193] Kortaberria G. Characterization of Large Machine Tool Volumetric Behaviour in Workshop. https://www.ptb.de/emrp/fileadmin/documents/tim/tim-sharepoint/Meetings/2016-05-18%20Final%20Workshop/Presentations/11_TIM_Workshop_Kortaberria_IK4-TEKNIKER.pdf.

[194] Wennemer M. A Case Study on a Very Large Machine Tool: Systematic Deviations and Thermo-Elastic Deformations. http://downloads.etalon-ag.com/Seminar%20Flyer_en%20Druck.pdf.

[195] (2014). Good Practice Guide for Assessing the Fitness for Purpose for Dimensional Measurements on Machine Tool.

[196] Metrology Solutions for Productive Process Control. Renishaw. http://resources.renishaw.com/en/details/brochure-metrology-solutions-for-productive-process-control--47618.

